# Mining novel starch-converting Glycoside Hydrolase 70 enzymes from the Nestlé Culture Collection genome database: The *Lactobacillus reuteri* NCC 2613 GtfB

**DOI:** 10.1038/s41598-017-07190-z

**Published:** 2017-08-30

**Authors:** Joana Gangoiti, Sander S. van Leeuwen, Xiangfeng Meng, Stéphane Duboux, Christina Vafiadi, Tjaard Pijning, Lubbert Dijkhuizen

**Affiliations:** 1 Microbial Physiology, Groningen Biomolecular Sciences and Biotechnology Institute (GBB), University of Groningen, Nijenborgh 7, 9747 AG Groningen, The Netherlands; 2 Nestlé Research Center, Vers-Chez-Les-Blanc, Lausanne, Switzerland; 3 Biophysical Chemistry, Groningen Biomolecular Sciences and Biotechnology Institute (GBB), University of Groningen, Nijenborgh 7, 9747 AG Groningen, The Netherlands; 4 CarbExplore Research BV, Zernikepark 12, 9747 AN Groningen, The Netherlands

## Abstract

The Glycoside hydrolase (GH) family 70 originally was established for glucansucrases of lactic acid bacteria (LAB) converting sucrose into α-glucan polymers. In recent years we have identified 3 subfamilies of GH70 enzymes (designated GtfB, GtfC and GtfD) as 4,6-α-glucanotransferases, cleaving (α1 → 4)-linkages in maltodextrins/starch and synthesizing new (α1 → 6)-linkages. In this work, 106 putative GtfBs were identified in the Nestlé Culture Collection genome database with ~2700 genomes, and the *L*. *reuteri* NCC 2613 one was selected for further characterization based on variations in its conserved motifs. Using amylose the *L*. *reuteri* NCC 2613 GtfB synthesizes a low-molecular-mass reuteran-like polymer consisting of linear (α1 → 4) sequences interspersed with (α1 → 6) linkages, and (α1 → 4,6) branching points. This product specificity is novel within the GtfB subfamily, mostly comprising 4,6-α-glucanotransferases synthesizing consecutive (α1 → 6)-linkages. Instead, its activity resembles that of the GtfD 4,6-α-glucanotransferases identified in non-LAB strains. This study demonstrates the potential of large-scale genome sequence data for the discovery of enzymes of interest for the food industry. The *L*. *reuteri* NCC 2613 GtfB is a valuable addition to the starch-converting GH70 enzyme toolbox. It represents a new evolutionary intermediate between families GH13 and GH70, and provides further insights into the structure-function relationships of the GtfB subfamily enzymes.

## Introduction

Lactic acid bacteria (LAB) are known to produce diverse extracellular polysaccharides (EPS) with applications in the food and health related industries^1–3^. Examples are the α-glucans that are synthesized by the action of a single glucansucrase (GS) enzyme from sucrose^4, 5^. These extracellular GS enzymes are exclusively found in LAB and initially were the only members belonging to the glycoside hydrolase family 70 (GH70). GH70 enzymes active on sucrose were proposed to share a common ancestor with the GH13 family enzymes, mainly acting on starch-like substrates. Both families share mechanistic, structural, and evolutionary characteristics^6–8^. Similar to GH13 family enzymes, GSs use an α-retaining double displacement mechanism, and possess an (β/α)_8_-barrel catalytic domain comprising an active site with 4 conserved sequence motifs (I–IV). However, GSs also have unique features that distinguish them from GH13 enzymes^9^. First, GSs display a rather unusual U-fold domain organization in which 4 of their 5 domains (domains A, B, IV and V) are formed by 2 discontinuous segments of polypeptide chains. Second, in addition to the core domains A, B and C, also present in GH13 enzymes, GSs possess domains IV and V that are absent in GH13 enzymes. Finally, in GSs the catalytic (β/α)_8_-barrel of domain A is circularly permuted compared with that of GH13 enzymes, and as a result, the order of the conserved sequence motifs in GSs is II-III-IV-I, different from the order I-II-III-IV found in GH13 enzymes^10–13^. Based on these observations it has been proposed that GSs evolved from GH13 α-amylases rather than vice versa, *via* gene duplication, truncation, and domain insertion events^10^.

The proposed evolution of the sucrose-acting GH70 from starch-acting GH13 enzymes was further supported by the discovery of three novel GH70 subfamilies of enzymes acting on starch-like substrates, but unable to use sucrose as substrate. First, the GtfB GH70 subfamily of enzymes showing a GS-like U-fold domain organization with a permuted catalytic (β/α)_8_-barrel, was identified in several *Lactobacillus* strains^14, 15^. The *Lactobacillus reuteri* 121 GtfB is the main representative of this GH70 subfamily, displaying 4,6-α-glucanotransferase (4,6-α-GTase) activity as it is cleaving (α1 → 4) linkages and forming new consecutive (α1 → 6) glucosidic linkages^14^. This main (α1 → 6) transfer activity results in the synthesis of isomalto-/malto-polysaccharides (IMMP), consisting of linear (α1 → 6) glucan chains attached to the non-reducing end of malto-oligosaccharides and/or starch fragments^16^. With the availability of the crystal structure of the *L*. *reuteri* 121 GtfB-ΔNΔV protein it was proposed that evolution from a starch-acting α-amylase to GSs occurred *via* a GtfB-like intermediate, as the active site of this enzyme shows structural similarity to both GH13 α-amylases and GH70 GSs^17^. Although the 4,6-α-GTase appeared to be the common activity within the GtfB GH70 subfamily, a GtfB-like enzyme displaying (α1 → 3) linkage specificity was recently identified in the *Lactobacillus fermentum* NCC 2970 genome and characterized^18^. The *L*. *fermentum* NCC 2970 GtfB showed unique variations in some of the residues in the conserved regions II and IV contributing to the active site donor/acceptor substrate binding subsites. The *L*. *fermentum* NCC 2970 GtfB activity results in the synthesis of a unique α-glucan with alternating (α1 → 3)/(α1 → 4)-linkages and with (α1 → 3,4) branching points. To date, the *L*. *fermentum* NCC 2970 GtfB is the only reported enzyme displaying 4,3-α-glucanotransferase activity in the GH70 family and GH-H clan. In addition to the GtfB type of enzymes, we have also identified interesting evolutionary intermediates between GH13 and GH70 families in non-LAB strains forming the new GtfC and GtfD GH70 subfamilies^19–21^. Rather surprisingly, these GtfC and GtfD enzymes display the non-permuted GH13 type of domain organization but with a continuous domain IV inserted in domain B. Characterization of the *Exiguobacterium sibiricum* 255–15 GtfC, *Azotobacter chroococcum* NCIMB 8003 GtfD and *Paenibacillus beijingensis* DSM 24997 GtfD enzymes revealed that they act as 4,6-α-GTases, in agreement with the clear sequence similarity shared between GtfB-like 4,6-α-GTases and GtfC-/GtfD-like enzymes in their motifs I to IV. Similar to the *L*. *reuteri* 121 GtfB enzyme, the *E*. *sibiricum* 255-15 GtfC catalyzes the cleavage of the (α1 → 4) linkages present in starch/maltodextrin substrates and the formation of new successive (α1 → 6) glucosidic linkages. The product specificity of the *A*. *chroococcum* NCIMB 8003 and *P*. *beijingensis* GtfD DSM 24997 enzymes, however, was found to be novel for the GH70 family, as both GtfD enzymes are unable to synthesize consecutive (α1 → 6) glucosidic linkages. Using amylose these GtfD enzymes form α-glucans with linear (α1 → 4) sequences interspersed with single (α1 → 6) glucosidic linkages and (α1 → 4,6) branching points. Interestingly, the structure of these polymers resembles that of the reuteran polymer synthesized by the *L*. *reuteri* 121 GtfA GS from sucrose, regarded as a potentially valuable dietary fiber^22^, which improves the quality of bread^23^.

Besides contributing to the understanding of the evolutionary history of GH70 proteins, the discovery of the GtfB, GtfC and GtfD subfamilies of GH70 enzymes has opened new perspectives for the production of novel dietary fibers and/or slowly digestible carbohydrates from starch. *In vitro* digestibility studies simulating the human gastrointestinal tract revealed that also the IMMP produced by the *L*. *reuteri* 121 GtfB have a high dietary fiber content^16^. The direct action of the *P*. *beijingensis* DSM 24997 and *A*. *chroococcum* NCIMB 8003 GtfD enzymes on wheat starch also resulted in products less susceptible to *in vitro* digestion^21^. Such slowly digestible carbohydrates and dietary fibers are considered beneficial for human health.

In this work, the Nestlé Culture Collection (NCC) genome database was screened for genes encoding starch-converting GH70 enzymes. This database contains more than 2700 isolates, mostly LAB, of interest for the food industry. A total of 106 putative GtfB-like enzymes were identified, mostly present in *Lactobacillus* strains, further increasing the number of available GtfB-like protein sequences. One of these GtfB-like enzymes, showing unique further variations in the conserved regions I–IV and encoded by the probiotic bacterium *L*. *reuteri* NCC 2613, was selected for further characterization. Notably, 3D modeling of the *L*. *reuteri* NCC 2613 GtfB structure suggested that this enzyme lacks the tunnel-shaped active site observed in the *L*. *reuteri* 121 GtfB structure [17], most likely resulting in a more open architecture of the active site. In agreement with these differences, our data shows that the *L*. *reuteri* NCC 2613 GtfB converts amylose into a branched, low molecular mass (LMM) reuteran-like polymer. This reaction and product specificity resembles that of the GtfD enzymes found in non-LAB, but it is new within the GtfB-like GH70 subfamily. These results demonstrate the power of data generated from genome sequencing projects allowing a further expansion of the enzymatic tools that food technologists have for the reduction of the glycaemic index of starch-containing foods.

## Materials and Methods

### Origin of bacterial strains

*Escherichia coli* DH5α (Phabagen) was used as host for cloning purposes. *Escherichia coli* BL21 Star (DE3) (Invitrogen) was used for *Lactobacillus reuteri* NCC 2613 GtfB protein expression. Other bacteria used in this study originate from the Nestlé Culture Collection and can be obtained by contacting the Nestlé Culture Collection coordinator Stéphane Duboux (stephane.duboux@rdls.nestle.com). The *L*. *reuteri* NCC 2613 has been deposited at the French National Collection of Microorganism Cultures (CNCM) as CNCM I-2452 and can be obtained under the Budapest treaty conditions.

### Identification of new GtfB-like proteins in the Nestlé Culture Collection genome database

Annotation of the GH70 family enzymes present in the NCC genome database was performed using the dbCAN database for automated Carbohydrate-active enzyme ANnotation^24^. Hits having an E-Value below 1E-5 and a bit score above 350 were considered. As a result 788 protein sequences were retrieved and used together with the *L*. *reuteri* 121 GtfB (Accession number: AAU08014.2), *Leuconostoc citreum* NRRL B-1299 branching sucrase (Accession number: CDX66820.1) and *L*. *reuteri* 180 Gtf180 GS (Accession number: AAU08001.1) protein sequences for the construction of multiple sequence alignments with Jalview 2 desktop application using the MUSCLE algorithm^25^. Sequences were only considered to be putative starch-acting GH70 enzymes if they possessed an aromatic Tyr (Y1055 *L*. *reuteri* 121 GtfB numbering) replacing the conserved Trp typically present in GSs, resulting in a set of 106 GtfB-like gene products^26^. Branching sucrases were distinguished by the presence of a Gly residue at this position in the alignments^27^. For further analysis, the set of GtfB proteins identified within the NCC genome database was expanded with characterized GH70 proteins indexed in CAZy (http://www.cazy.org/) and aligned by MUSCLE, using default parameters. A phylogenetic tree was obtained by the Maximum Likelihood method based on the JTT matrix model using MEGA6^28^. The analysis involved 167 protein amino acid sequences. Partial deletion of the positions containing alignment gaps and missing data was conducted. Statistical confidence of the inferred phylogenetic relationships was assessed by performing 1,000 bootstrap replicates. The GenBank accession numbers of the GH70 proteins used in this section are provided in Table S1.

### Analysis of the *L*. *reuteri* NCC 2613 GtfB protein sequence

Multiple amino acid sequence alignments were generated with Clustal Omega (http://www.ebi.ac.uk/Tools/msa/clustalo/) and visualized by using the Jalview 2 desktop application. Subcellular localization of the *L*. *reuteri* GtfB protein was predicted using CELLO v.2.5: subCELlular LOcalization predictor (http://cello.life.nctu.edu.tw/) and its theoretical *M*_w_ (molecular weight) was predicted by ExPASy Compute pI/*M*_w_ (http://web.expasy.org/compute_pi/).

### Structural modelling of the *L*. *reuteri* NCC 2613 GtfB protein

A three-dimensional model of the *L*. *reuteri* NCC 2613 GtfB was constructed with Phyre^29^, using the recently determined three-dimensional structure of *L*. *reuteri* 121 GtfB 4,6-α-GTase (PDB ID: 5JBD; ^17^) as a template for one-to-one threading of the full-length sequence, with default settings. For comparison of binding sites, also the crystal structures of *L*. *reuteri* 121 GtfB 4,6-α-GTase complexed with maltopentaose or 6^4^-α-D-glucosyl-maltotetraose, which is an isomalto-/maltooligosaccharide transglycosylation product resulting from GtfB activity on maltohexaose (PDB ID: 5JBE, 5JBF) were used.

### Cloning of the *L. reuteri* NCC 2613 gtfB gene

The *gtfB* gene fragment encoding for an N-terminally truncated variant of the GtfB protein (GtfB-ΔN) was amplified from *L*. *reuteri* NCC 2613 genomic DNA with Phusion DNA polymerase (Finnzyme, Helsinki, Finland) and cloned into a modified pET15b vector by ligation-independent cloning (LIC)^30^. The primers used contained LIC-compatible extensions (underlined), and were: Forward CAGGGACCCGGTGGGCATTTACTTGGAAATC and Reverse CGAGGAGAAGCCCGGTTAATCGTCTTCAATATTAGC. The KpnI-digested vector and the generated PCR product were purified from gel, and subsequently treated with T4 DNA polymerase in the presence of dATP and dTTP, respectively. The two reaction products were mixed together in a 1:4 molar ratio, and the mixture was used to transform chemical- competent *Escherichia coli* DH5α cells, yielding pET15b*/gtfB-ΔN*. This vector encodes the GtfB-ΔN protein (amino acids 417 to 1281) fused with an N-terminal His6-tag cleavable by a 3 C protease. The constructed expression vector pET15b*/gtfB-ΔN* was verified by nucleotide sequencing (GATC, Cologne, Germany), and transformed into *E*. *coli* BL21 Star (DE3).

### Expression and purification of the *L*. *reuteri* NCC 2613 GtfB protein

Fresh Luria Broth medium supplemented with ampicillin (100 μg ml^−1^) was inoculated with 1% (v/v^−1^) of an overnight culture of *E. coli* BL21 Star (DE3) harboring the pET15b/gtfB-ΔN plasmid, and cultivated at 37 °C and 160 rpm. Protein expression was induced at an OD600 of 0.7 by adding isopropyl-β-d-1-thiogalactopyranoside to 0.1 mM, and cultivation was continued for 20 h at 16 °C. Cells were harvested by centrifugation (10,000 × g, 20 min). The GtfB-ΔN enzyme was purified by Ni^2+^-nitrilotriacetic acid (NTA) affinity chromatography (Sigma Aldrich, St. Louis, USA) as described previously20. Purity was assessed by SDS-PAGE analysis, and protein concentrations were determined by measuring the absorbance at 280 nm, using a NanoDrop 2000 spectrophotometer (Isogen Life Science, De Meern, The Netherlands).

### Enzyme activity assays

The total activity of the *L*. *reuteri* NCC 2613 GtfB-ΔN enzyme (initial rate) was determined by the amylose-iodine staining method using 0.125% (w v^−1^) amylose V (AVEBE, Foxhol, The Netherlands) as described before^19, 31^. Routinely, enzymatic assays were performed with 2 μg ml^−1^ of enzyme in 25 mM sodium acetate (pH 5.5) and 1 mM CaCl_2_. The decrease in absorbance of the α-glucan-iodine complex resulting from transglycosylation and/or hydrolytic activity was monitored at 660 nm for 8 min at 40 °C. One unit of activity was defined as the amount of enzyme converting 1 mg of substrate per min. The pH profile and optimum pH were determined at 40 °C by varying the pH between 3.0 and 10.0. Sodium citrate buffer (25 mM) was used at pH 3.0–7.0, sodium phosphate buffer (25 mM) at pH 7.0–8.0, Tris-HCl (25 mM) at pH 8.0–9.0, and sodium bicarbonate (25 mM) at pH 9.0–10.0.

### Substrate specificity of the *L*. *reuteri* NCC 2613 GtfB enzyme

The substrate specificity of the *L. reuteri* NCC 2613 GtfB enzyme was investigated by incubating 40 μg ml^−1^ of purified enzyme with either 25 mM sucrose (Acros), nigerose (Sigma-Aldrich), panose (Sigma-Aldrich), isomaltose (Sigma-Aldrich), isomaltotriose (Sigma-Aldrich), isomaltopentaose (Carbosynth), malto-oligosaccharides (MOS) with degrees of polymerization (DP) 2–7 (Sigma-Aldrich), or with 0.6% (w v^−1^) amylose V (AVEBE, Foxhol, The Netherlands), potato starch (Sigma-Aldrich) or amylopectin (Sigma-Aldrich). Potato starch was pregelatinized by autoclaving (15 min, 120 °C). Amylose V (1%, w/v) was prepared as a stock solution in sodium hydroxide (1 M). Prior to use, the stock solution was neutralized with 7 M HCl and diluted to a concentration of 0.85% (w v^−1^). Incubations were carried out in 25 mM sodium acetate buffer, pH 5.5 with 1 mM CaCl_2_ at 37 °C for 24 h. Reactions were stopped by heating the samples to 100 °C for 8 min. The progress of the reactions was analysed by thin-layer chromatography (TLC) and/or high-performance-anion-exchange chromatography (HPAEC).

### Thin Layer Chromatography and High Performance Anion Exchange Chromatography with pulsed amperometric detection analysis

Carbohydrate samples were spotted in 1-cm lines on a TLC silica gel 60F254 sheet (Merck, Darmstadt, Germany). The TLC plate was run for 6 h in butanol:acetic acid:water (2:1:1, v v^−1^), and products were visualized with orcinol/sulfuric acid staining. A mixture of glucose and malto-oligosaccharides (DP2 to DP7) was used as standard.

HPAEC-PAD analysis was performed using an ICS3000 workstation (Thermo Scientific, Amsterdam, The Netherlands), equipped with a CarboPac PA-1 column (Thermo Scientific; 250 × 2 mm) and an ICS3000 electrochemical detection module. Prior to analysis the carbohydrate samples were diluted 1:300 in DMSO and the oligosaccharides were separated at a 0.25 ml min^−1^ flow rate by using a sodium acetate gradient (10 to 240 mM) in 100 mM NaOH over 57 min. The injection volume of each sample was 5 μl. The identity of the peaks was determined using commercial oligosaccharide standards and a mixture of MOS of DPs from 2 to 30.

### HPSEC analysis

Molecular mass distribution of the product mixtures was determined using a size exclusion chromatography system (Agilent Technologies 1260 Infinity) equipped with a multi angle laser light scattering detector (SLD 7000 PSS, Mainz), a viscometer (ETA-2010 PSS, Mainz) and a differential refractive index detector (G1362A 1260 RID Agilent Technologies), as described before^20, 31^. Briefly, samples were dissolved at a concentration of 4 mg ml^−1^ in DMSO-LiBr (0.05 M) and separation was carried out by using three PFG-SEC columns with porosities of 100, 300 and 4000 Å, coupled with a PFG guard column. The eluent was DMSO-LiBr (0.05 M) at a flow rate of 0.5 ml min^−1^. The system was calibrated and validated using a standard pullulan kit (PSS, Mainz, Germany) with *M*_w_ ranging from 342 to 805 000 Da. The specific RI increment value (*dn*/*dc*) was also measured by PSS and was 0.072 ml g^−1^ (personal communication with PSS). The multiangle laser light scattering signal was used to determine the molecular masses of amylose V and the high molecular mass (HMM) polysaccharides generated by the *A*. *chroococcum* and *P*. *beijingensis* GtfD enzymes. The *dn*/*dc* values for these polysaccharides were taken to be the same as for pullulan. The molecular masses of the *L*. *reuteri* NCC 2613 GtfB, *L*. *reuteri* 121 GtfB and *P*. *beijingensis* GtfD low molecular mass (LMM) polymers were determined by universal calibration method. Measurements were performed in duplicate.

### Synthesis, isolation and structural analysis of the products from amylose V incubation with *L*. *reuteri* NCC 2613 GtfB

Incubations of amylose V (0.6%wv^−1^) and GtfB-ΔN (0.2 mg) were performed under the conditions described in “Substrate specificity of the *L*. *reuteri* NCC 2613 GtfB”. After incubation for 24 h at 37 °C, the reaction was stopped by transfer to 100 °C for 10 min. The polysaccharide was separated from trace amounts of small oligosaccharides (DP < 5) also present in the product mixture by size-exclusion chromatography on a Biogel P2 column (2.5 × 50 cm; Bio-Rad, Veenendaal, The Netherlands) using 10 mM NH_4_HCO_3_ as eluent at a flow rate of 48 ml h^−1^.

#### NMR spectroscopy

Resolution-enhanced 1D/2D ^1^H and ^13^C NMR spectra were recorded in D_2_O on a Varian Inova-500 spectrometer (NMR center, University of Groningen, The Netherlands) at a probe temperature of 298 K. Samples were exchanged twice in D_2_O (99.9 at% D, Cambridge Isotope Laboratories, Inc., Andover, MA) with intermediate lyophilization, and then dissolved in 0.6 ml of D_2_O. One-dimensional 500-*MHz* ^1^H NMR spectra were recorded at a 4000 *Hz* spectral width and 16k complex points, using a WET1D pulse to suppress the HOD signal. Two-dimensional ^1^H-^1^H spectra (COSY, TOCSY MLEV17 30, 50, and 150 ms, and ROESY 300 ms) were recorded with 4000 *Hz* spectral width, collecting 200 increments. In case of TOCSY spectra 2000 complex data points were collected, for COSY and ROESY spectra 4000 complex data points were used. 2D ^13^C-^1^H NMR spectra were recorded in 128 increments of 2000 complex points with 4000 *Hz* spectral width in *t2* and 10 000 *Hz* in *t1*. The data were processed using MestReNova 5.3 (Mestrelabs Research SL, Santiago de Compostella, Spain). Manual phase correction and Whittacker smoother baseline correction were applied to all spectra. Chemical shifts (*δ*) are expressed in ppm with reference to internal acetone (*δ* 2.225 for ^1^H and *δ* 31.08 for ^13^C).

#### Methylation analysis

Polysaccharide samples (~5 mg) were per-methylated using CH_3_I and solid NaOH in DMSO, as described before^32^. After hydrolysis with 2 M trifluoroacetic acid (2 h, 120 °C), the partially methylated monosaccharides generated were reduced with NaBD_4_ (2 h, room temperature, aqueous solution), and the solution was neutralized with acetic acid. Subsequently, boric acid was removed by co-evaporation with methanol. The resulting partially methylated alditols were per-acetylated using pyridine:acetic anhydride (1:1 v/v) at 120 °C yielding mixtures of partially-methylated alditol acetates, which were analyzed by GLC-EI-MS as described^32^.

#### Enzymatic treatments with α-amylase, dextranase and pullulanase

The α-glucan samples (5 mg) were dissolved in 500 μl of sodium acetate buffer (50 mM pH 5.0), and incubated separately with excess amounts of α-amylase (*Aspergillus oryzae*; Megazyme), dextranase (*Chaetomium erraticum*; Sigma-Aldrich), and pullulanase M1 (*Klebsiella planticola*; Megazyme) at 37 °C. After 48 h, the degree of hydrolysis was evaluated by TLC and/or HPAEC. Starch, dextran and pullulan, were used as positive controls for the α-amylase, dextranase and pullulanase treatments, respectively, obtaining fully hydrolyzed products under these conditions.

### Data and materials Availability

All relevant data are within the paper and its Supporting Information files.

## Results and Discussion

### Identification of novel starch active GH70 enzymes within the NCC genome database

The NCC genome database with mostly lactic acid bacteria (LAB) was screened for novel GtfB-like enzymes. Analysis by dbCAN resulted in the identification of 788 sequences as GH70 family proteins. Out of 106 of these proteins were annotated as putative GtfB proteins sharing more than 70% identity with the N-terminally truncated *L*. *reuteri* 121 GtfB enzyme. Also the recently characterized *L*. *fermentum* 4,3-α-GTase was identified in this NCC database^18^. As reported before, most of the GtfB proteins present in the NCC were encoded by *Lactobacillus* strains^26^, with the exception of the 3 GtfB-like proteins found in *Leuconostoc citreum* NCC 2514 and *Streptococcus thermophilus* strains NCC 903 and NCC 2408. Among the GH70 family protein sequences, 519 GSs, 35 branching sucrases, and 36 bifunctional GH70 enzymes containing two catalytic domains (GS and branching sucrase) were also identified. The GtfB type of gene is distributed in 103 of the 1101 sequenced *Lactobacillus* genomes, but only in 2 and 1 of the 360 and 141 sequenced *Streptococcus* and *Leuconostoc* strains, respectively. These 590 sucrose-active GH70 enzymes were found more widespread in different LAB species, in *Lactobacillus*, *Oenococcus*, *Pediococcus*, *Streptococcus*, *Leuconostoc* and *Weissella*. Interestingly, GSs were found to be distributed throughout these genera, while branching sucrases and bifunctional GH70 enzymes were only found in *Leuconostoc* species. The other 92 proteins were partial enzymes lacking motifs II and III and could therefore not be correctly attributed to one of the above categories. No GtfC or GtfD proteins were found in the NCC genome database, reflecting its strong bias towards LAB strains.

**Figure 1:**
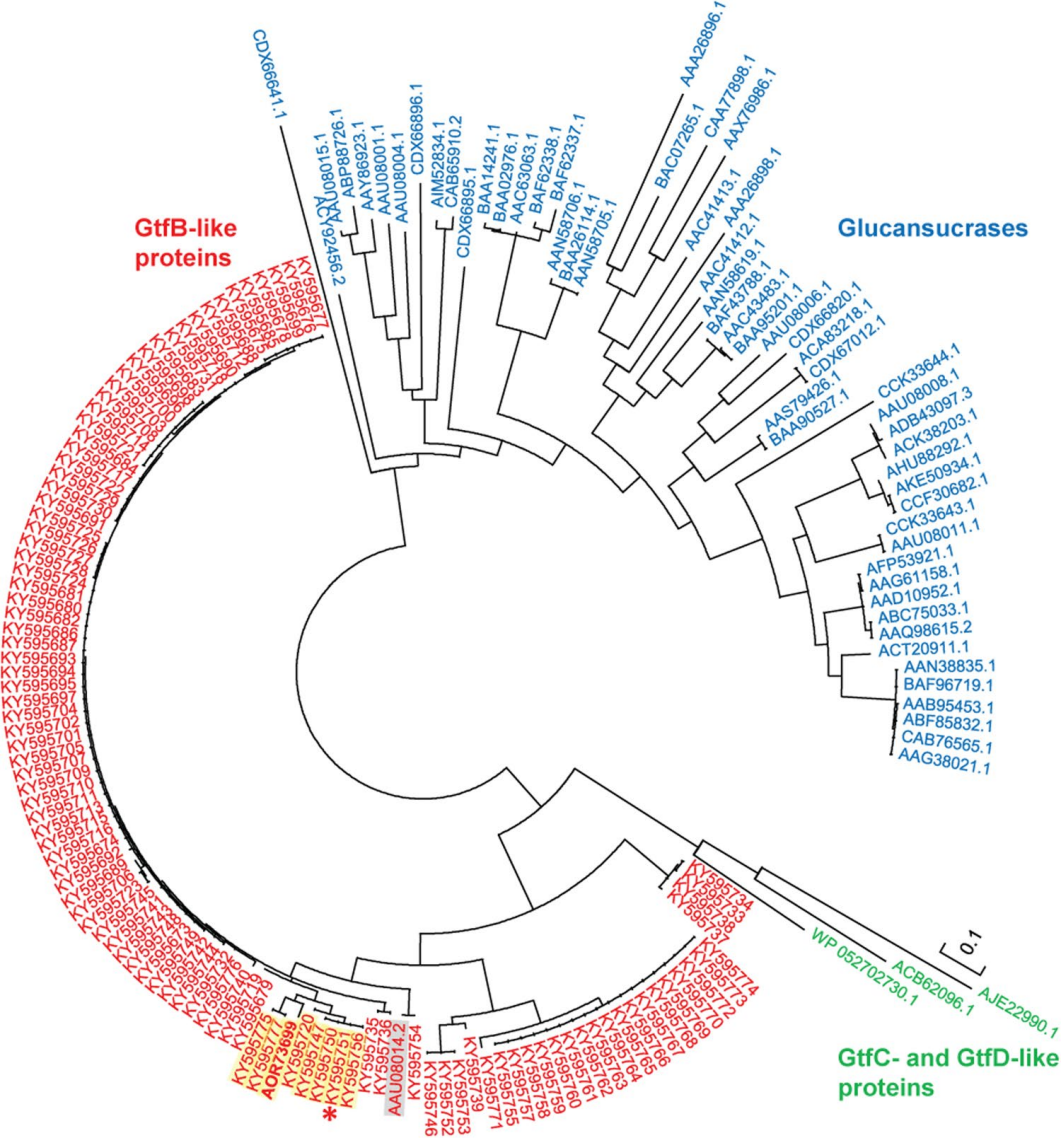
Phylogenetic tree constructed using the complete amino acid sequences of the characterized GH70 proteins annotated in the CAZy database, and of (putative) GH70 GtfB-like proteins identified in the NCC genome database. The evolutionary history was inferred by using the Maximum Likelihood method based on the JTT matrix-based model. The bar represents a genetic distance of 0.1 substitutions per position (10% amino acid sequence difference). The protein sequences are annotated by their Genbank accession number. The *L*. *reuteri* 121 GtfB 4,6-α-GTase is highlighted with a grey background. The *L*. *fermentum* GtfB 4,3-α-GTase is shown in bold. The *L*. *reuteri* NCC 2613 GtfB is indicated by an asterisk. The GtfB enzymes showing differences in functionally important residues in motifs II and IV are highlighted in yellow.

A phylogenetic tree showing the evolutionary relationships between the 106 putative GtfB proteins and a selection of the biochemically characterized GH70 proteins is depicted in Fig. 1. These GtfB enzymes clearly grouped in the GtfB-like GH70 subfamily, which is located in between the GSs and the GtfC/GtfD subfamilies as reported before^19, 20, 26^.

**Figure 2:**
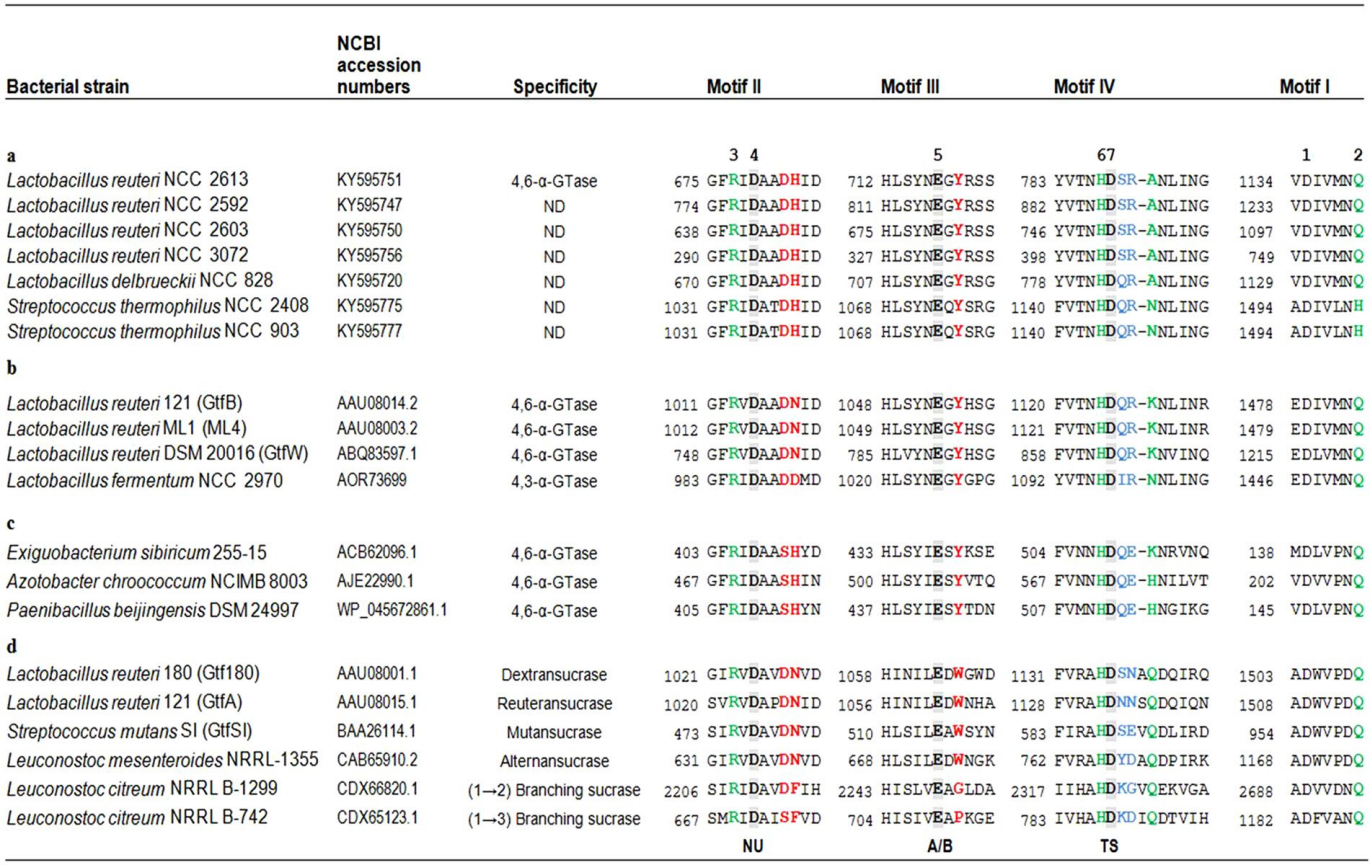
Sequence alignment of conserved motifs I-IV in the catalytic domain of putative GtfB-like proteins identified in the NCC genome database and other GH70 starch and sucrose acting enzymes: (**a**) (Putative) GtfB-like enzymes showing differences in some of the residues in motifs II and IV forming the substrate-binding site, (**b**) Characterized GtfB-like enzymes, (**c**) Characterized GtfC-like and GtfD-like 4,6-α-GTase enzymes, (**d**) Characterized sucrose-active GSs enzymes. The seven strictly conserved amino acid residues in GH70 enzymes (indicated by the numbers 1 to 7 above the sequences) are also conserved in the GtfB-like proteins identified in the NCC genome database. Amino acids that constitute the catalytic triad are in bold and slightly shaded. Residues forming acceptor-binding subsites −1, + 1, and + 2 in Gtf180-ΔN are highlighted in green, red and blue, respectively. Symbols: NU, nucleophile; A/B, general acid/base; TS, transition state stabilizer.

With the aim of finding novel enzymes for the production of structurally different starch-derived dietary fibers, the conserved motifs I to IV of these 106 putative GtfB-like proteins were analyzed in detail (Fig. 2). Motifs I to IV of the GtfB enzymes identified in the NCC genome database displayed clear similarity with those corresponding to previously characterized 4,6-α-GTases, and were easily identified. The order of these conserved regions I to IV in the 106 identified GtfB-like sequences is II-III-IV-I, reflecting their circularly permutated domain organization. The seven highly conserved residues among these GH70 motifs, including the catalytic residues (D1015, E1053, D1125; *L*. *reuteri* 121 GtfB numbering) and residues involved in the formation of subsite -1 (R1013, H1124, D1479 and Q1484) were found in most of the identified GtfB-like protein sequences (except for two putative GtfB enzymes identified in *S*. *thermophilus* strains NCC 903 and NCC 2408). Six of these residues also are highly conserved within GH13 enzymes with the exception of Gln1484 (GtfB, *L*. *reuteri* 121 numbering), which is replaced by a His residue in GH13 enzymes (His140, BSTA *B*. *stearothermophilus* α-amylase numbering). Similar to GH13 enzymes the putative GtfB enzymes encoded by *S*. *thermophilus* strains NCC 903 and NCC 2408 have a His at this position. This difference supports the proposed evolution of GH70 GSs from GH13 α-amylases *via* a GtfB-like intermediate^17^, which will be further explored in future work.

Regarding other functionally important positions in these motifs, seven of the putative GtfB protein sequences identified in the NCC genome database show variations in some of the residues in motifs II and IV not previously observed in characterized GtfB 4,6-α-GTases. Four of these proteins are encoded by different *L*. *reuteri* strains (NCC 2592, NCC 2603, NCC 2613 and NCC 3072) isolated from small domestic animals (cat, dog and chicken), while three are encoded by dairy originating strains (two from *S*. *thermophilus* NCC 2408 and NCC 903 and one from *L*. *delbrueckii* NCC 828). Interestingly, in these seven GtfB proteins the conserved subsite + 1 Asn residue in motif II (N1029 in *L*. *reuteri* Gtf180 GS) is replaced by His, as in the case of GtfC and GtfD enzymes. This subsite + 1 Asn residue was found to be critical for the activity and linkage specificity of the Gtf180 GS^10^. Besides, variations were observed in the amino acids following the putative transition state stabilizer in motif IV. Specifically, the four GtfB enzymes encoded by different *L*. *reuteri* strains have a Ser and Ala at positions 1137 and 1140 (Gtf180 *L*. *reuteri* 180 numbering), instead of the conserved Gln and Lys typically found in most GtfB-and GtfC-like 4,6-α-GTases. The conserved Lys at position 1140 is also replaced in the two GtfB enzymes identified in *S*. *thermophilus* strains, as well as in the GtfB of L. *delbrueckii* NCC 828, by Asn and Ala, respectively. Previous mutational studies combined with structural data revealed that these residues contribute to glycosidic linkage specificity in GSs^33–35^. In case of the GtfD 4,6-α-GTase enzymes synthesizing reuteran-like polymers, the Gln residue at position 1137 is also conserved, whereas the Lys residue at position 1140 is substituted by a His and was proposed to define this novel product specificity^21^ It is noteworthy that the *L*. *fermentum* GtfB, which shares high identity with *L*. *reuteri* 121 GtfB but displays 4,3-α-GTase activity, also contains unique variations in residues 1029, 1137 and 1140, providing further support for the suggestion that these are “hot-spot” positions for product specificity in GtfB enzymes^18, 19, 21^. Phylogenetically, these seven putative GtfB enzymes and the *L*. *fermentum* GtfB 4,3-α-GTase are closely related, as they are more than 82% identical (Fig. 1). We decided to analyze in more detail the *L*. *reuteri* NCC 2613 GtfB, as representative of these seven new GtfB enzymes identified in the NCC genome database, displaying unique variations in their homology motifs.

### Amino acid sequence analysis and structure modelling of the GtfB-like enzyme of *L*. *reuteri* NCC 2613

The *L*. *reuteri* NCC 2613 genome contains a single gene coding for a putative GH70 enzyme of 1281 amino acids with a theoretical molecular mass of 145 kDa. As reported for other GH70 family proteins, the *L*. *reuteri* NCC 2613 GH70 enzyme is predicted to function as an extracellular protein. Alignment of its amino acid sequence with biochemically characterized GH70 enzymes showed highest sequence identity with the *L*. *fermentum* GtfB 4,3-α-GTase (83% identity), a fact reflected in their close distance in the phylogenetic tree (Fig. 1). The characterized GtfB 4,6-α-GTase enzymes of *L*. *reuteri* 121, *Lactobacillus reuteri* ML1 and *Lactobacillus reuteri* DSM 20016 also share significant amino acid identity (76%, 75% and 66% identity) with the *L*. *reuteri* NCC 2613 GH70 enzyme, further suggesting that also this protein belongs to the GtfB subfamily of GH70 enzymes.

**Figure 3:**
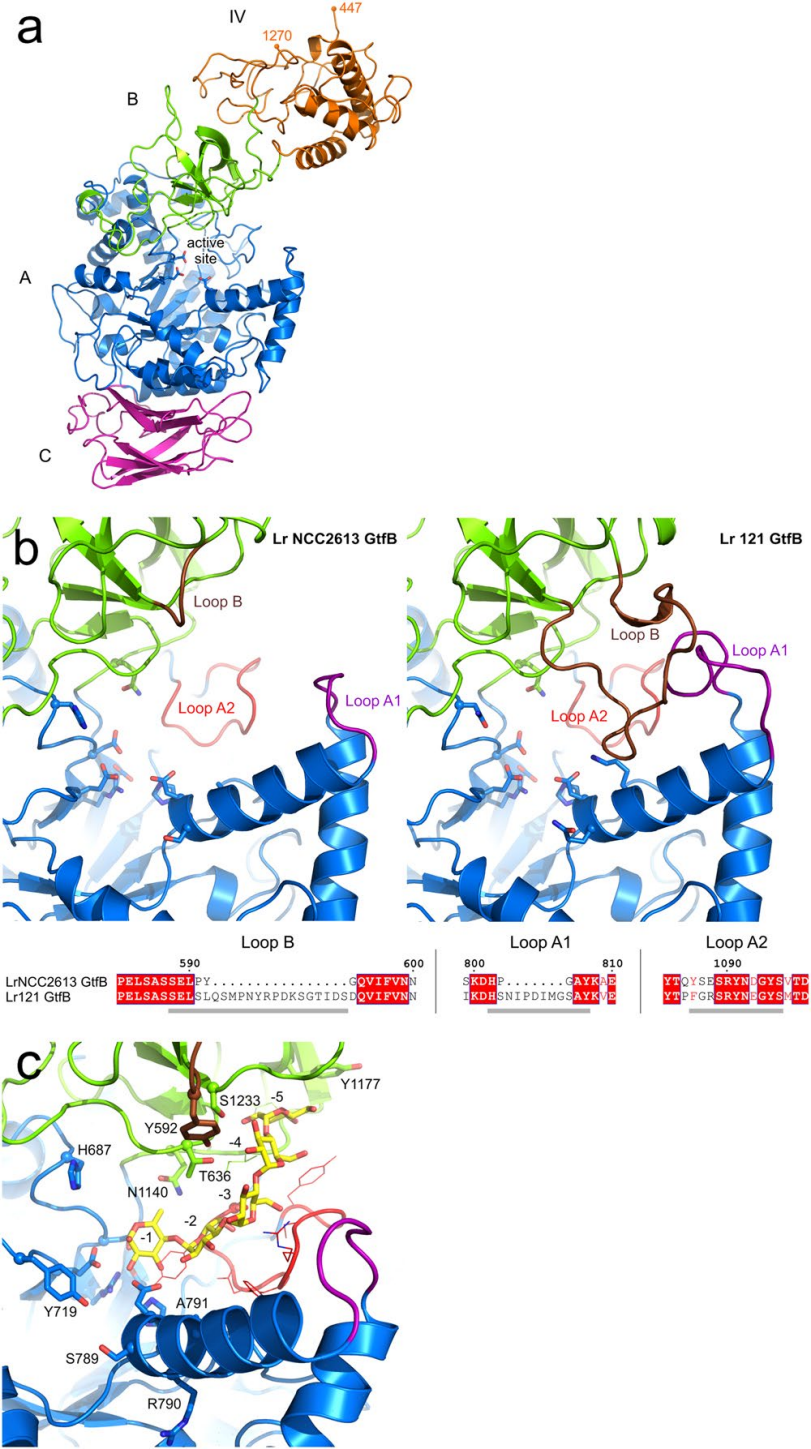
Homology model for *L*. *reuteri* NCC 2613 GH70 enzyme. Tertiary structure prediction was accomplished by using the Phyre2 server and the *L*. *reuteri* 121 GtfB-ΔNΔV as template^17^. (**a**) Overall 3D model structure of *L*. *reuteri* NCC 2613 GH70 enzyme. Domains A, B, C, IV and V are depicted in blue, green, magenta, yellow and red, respectively; the proposed catalytic residues in the active site are shown in stick representation (**b**) Close-up of the active site regions of the *L*. *reuteri* NCC 2613 GtfB enzyme and the *L*. *reuteri* 121 GtfB with loops A1, A2 and B highlighted; the sequence alignment of these loops in the two enzymes is also shown. In *L*. *reuteri* NCC 2613 GtfB, the much shorter loops A1 and B predict a much more open substrate binding groove than observed in the *L*. *reuteri* 121 GtfB enzyme. (**c**) Superposition of the maltopentaose bound in subsites −1 to −5 of the *L*. *reuteri* 121 GtfB (PDB ID: 5JBF^17^) with the *L*. *reuteri* NCC 2613 GtfB model. Residues near the binding groove are indicated.

The obtained 3D model of *L*. *reuteri* NCC 2613 GH70 enzyme, based on the *L*. *reuteri* 121 GtfB-ΔNΔV 4,6-α-GTase crystal structure^17^, comprises domains A, B, C and IV (Fig. 3a); it reflects the high sequence similarity between the two enzymes (79% identity for these domains). The same “U-fold” domain organization is observed, with a circularly permuted catalytic (β/α)_8_ barrel in domain A, characteristic of GSs and GtfB type of enzymes. Sequence comparison revealed that the *L*. *reuteri* NCC 2613 GH70 enzyme, like the *L*. *reuteri* 121 GtfB enzyme, also has an N-terminal variable domain (residues 1-446) and lacks a C-terminal variable domain. In its catalytic domain (A), the spatial arrangement of the proposed catalytic residues in the active center (D679, E717 and D788, see Fig. 3b) is similar to that of *L*. *reuteri* 121 GtfB-ΔNΔV. On the other hand, notable structural differences were observed between the substrate binding sites of the two enzymes. Most importantly, whereas the *L*. *reuteri* 121 enzyme features a tunnel extending beyond the active site formed by the 13-residue loop A1 and the 20-residue loop B, the corresponding loops in the *L*. *reuteri* NCC 2613 GH70 enzyme are only 6 and 4 residues long (802–807 and 590–593, respectively; Fig. 3b). As a consequence, these loops do not form a tunnel covering donor substrate binding subsites, and the binding groove is fully accessible, like in α-amylases. Superposition with the maltopentaose-bound *L*. *reuteri* 121 GtfB-ΔNΔV structure showed that residues in the highly similar loop A2 of the *L*. *reuteri* NCC 2613 GH70 enzyme likely interact with bound substrates, and so may residue Y592 from loop B (Fig. 3c). Also its tyrosine residue (Y1177, corresponding to Y1521 of the *L*. *reuteri* 121 GtfB enzyme) at subsite −6 is conserved (not shown) to provide an aromatic stacking interaction. Other notable differences are the presence of a histidine residue (H683) in motif II replacing the asparagine present in 4,6-α-GTases (N1019 in *L*. *reuteri* 121 GtfB), and the three residues following the transition state stabilizer in motif IV (SRA replacing QRK). On the other hand, its tyrosine residue near subsite + 1 (Y719, motif III) is conserved with 4,6-α-GTases. Residues from these motifs are known to contribute to the product specificity of GH70 enzymes. These structural differences observed in the architecture of the active site of the 3D model of the *L*. *reuteri* NCC 2613 GH70 enzyme prompted us to study the reaction and product specificity of this enzyme.

### Purification and biochemical properties of the *L*. *reuteri* NCC 2613 GH70 enzyme

Previous work showed that truncation of the N-terminal variable region of the *L*. *reuteri* 121 GtfB did not affect the enzyme catalytic properties, but facilitated protein expression^31^. Thus, the *L*. *reuteri* NCC 2613 gene encoding a putative GtfB enzyme was cloned and expressed in *E*. *coli* (DE3) BL21 star without its N-terminal variable region (amino acids 417 to 1281). Under the conditions used, high protein expression levels were observed in the soluble fraction (see Fig. S1 in the supplemental material), and following His tag affinity purification a total of ~50 mg of pure protein per liter of culture was obtained. SDS-PAGE analysis revealed a single protein band with an apparent molecular weight of ~100 kDa, which fits the predicted molecular mass deduced from its amino acid sequence (98 kDa).

The purified *L*. *reuteri* NCC 2613 GH70 enzyme was inactive with sucrose but active with maltodextrins/starch (see below), confirming its identity as a GtfB-ΔN enzyme. In order to determine the best conditions for subsequent reactions, the effects of pH on its enzyme activity were determined by using amylose V as substrate. This GtfB-ΔN enzyme showed its maximal activity at pH 5.5, but exhibited a broad pH tolerance, retaining more than 80% of this activity over a pH from 4 to 9 (Data not shown). This pH profile significantly differs from those reported for other GtfB enzymes, which showed significantly lower activities at basic pH values^18, 31^. The specific total activity value of the purified *L*. *reuteri* NCC 2613 GtfB-ΔN on 0.125% (w v^−1^) amylose in 25 mM citrate phosphate buffer, pH 5.5, containing 1 mM CaCl_2_ at 40 °C was 24 ± 0.6 U mg^−1^. This value is similar to the one reported for the *L*. *fermentum* GtfB-ΔN 4,3-α-GTase (22 U mg^−1^), but remarkably higher than that determined for the *L*. *reuteri* 121 GtfB 4,6-α-GTase, namely 2.8 U mg^−1^ (at 40 °C and pH 5.5 and 5.0, respectively)^18^.

### Substrate and product specificity of the *L*. *reuteri* NCC 2613 GtfB enzyme

**Figure 4:**
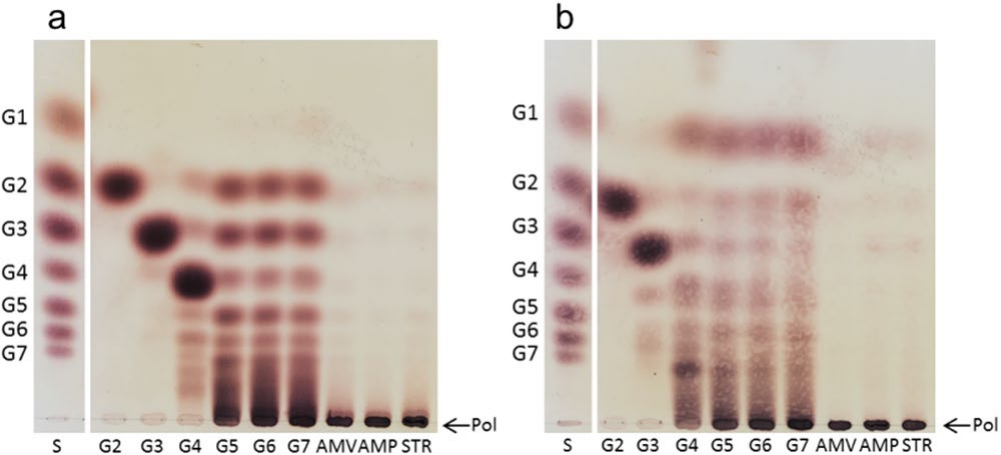
TLC analysis of the products produced by 40 μg ml^−1^ of the *L*. *reuteri* NCC 2613 GtfB-ΔN (**a**) and *L*. *reuteri* 121 GtfB (**b**) 4,6-α-glucanotransferase enzymes. Reaction mixtures containing 25 mM malto-oligosaccharides (DP2-DP7), 0.6% (w v^−1^) amylose V, 0.6% (w v^−1^) amylopectin, or 0.6% (w v^−1^) potato soluble starch were incubated at 37 °C and pH 5.5 (*L*. *reuteri* NCC 2613 GtfB) or pH 5.0 (*L*. *reuteri* 121 GtfB) during 24 h. S, standard; G1, glucose; G2, maltose; G3, maltotriose; G4, maltotetraose; G5, maltopentaose; G6, maltohexaose; G7, maltoheptaose; AMV, amylose V; AMP, amylopectin; STR, potato soluble starch; Pol, polymer.

The *L*. *reuteri* NCC 2613 GtfB-ΔN was incubated with different carbohydrate substrates at 37  °C for 24 h, and its activity was compared with that of the *L*. *reuteri* 121 4,6-α-GTase GtfB. As shown by TLC (Fig. 4), both GtfB enzymes displayed hydrolysis and transglycosylase (disproportionation) activity on MOS with DP4 to DP7, as revealed by the formation of a range of shorter and longer oligosaccharide products. Both enzymes also accumulated polymeric material from MOS. In case of the *L*. *reuteri* NCC 2613 GtfB-ΔN, polymer accumulation was detected when using maltopentaose (DP5) and longer MOS substrates, whereas the *L*. *reuteri* 121 GtfB already formed polymer from maltotetraose (DP4). Note that the *L*. *reuteri* 121 GtfB clearly accumulated glucose from the different MOS substrates. Instead, the *L*. *reuteri* NCC 2613 GtfB-ΔN accumulates maltose and some low molecular mass oligosaccharides, but not glucose as a side product of its hydrolase/transglycosidase activity. These observations suggest that these two GtfB enzymes differ in their mode of action. Incubation of amylose V, potato starch and amylopectin with the *L*. *reuteri* NCC 2613 GtfB-ΔN enzyme resulted in the appearance of some low molecular mass products that were not clearly detectable by TLC. Similar to the *L*. *reuteri* 121 GtfB, this *L*. *reuteri* NCC 2613 enzyme is also active on these polymeric substrates. As observed for other GH70 starch-modifying enzymes^14, 15, 18–21^, the *L*. *reuteri* NCC 2613 GtfB enzyme was inactive on sucrose, panose, nigerose, pullulan, dextran and isomalto-oligosaccharides with DP2, DP3, and DP5 (Data not shown).

**Figure 5:**
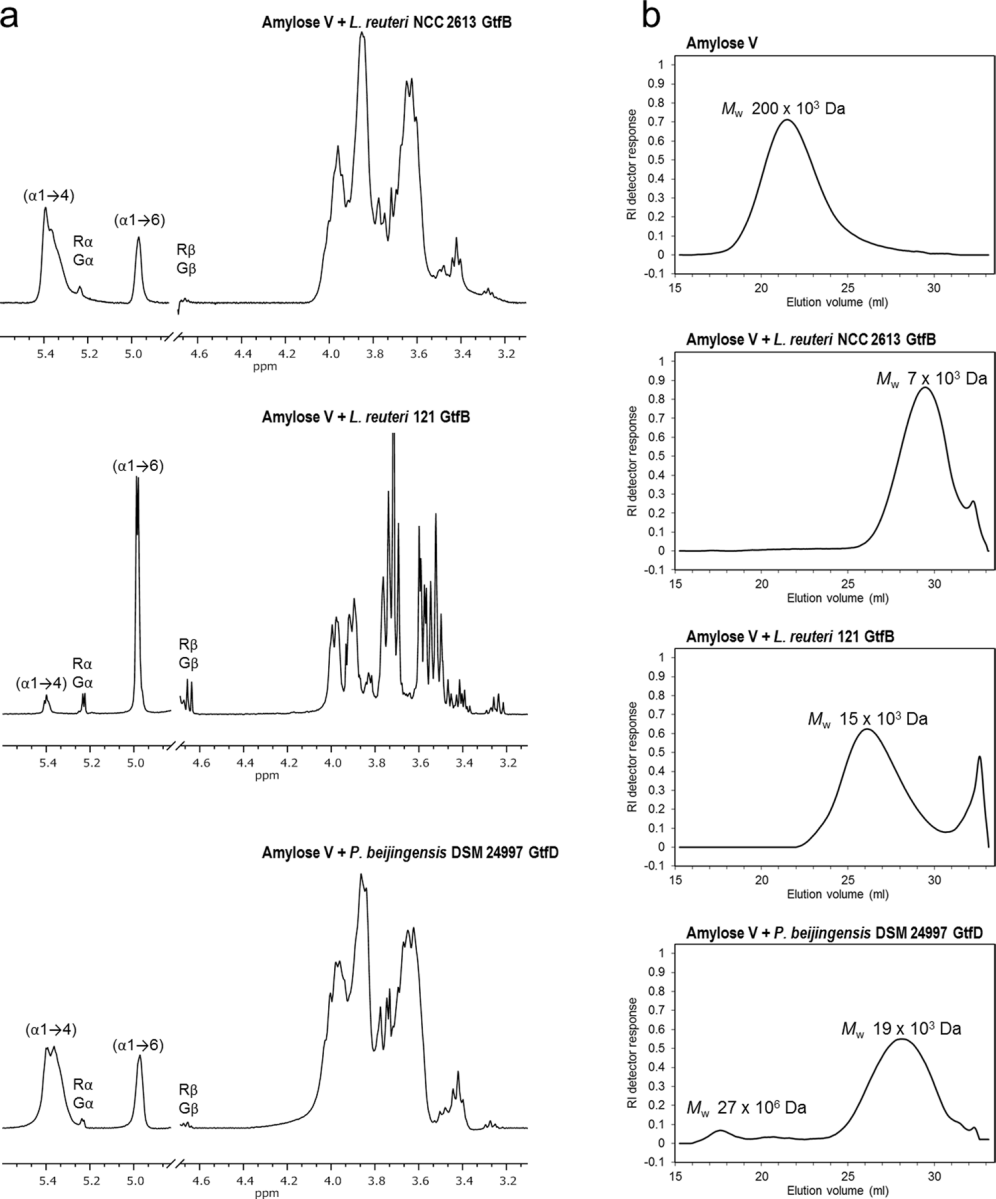
Characterization of the product mixtures formed by the incubation of 0.6% (w v^−1^) amylose V with 40 μg ml^−1^ of *L*. *reuteri* NCC 2613 GtfB-ΔN, *L*. *reuteri* 121 GtfB-ΔN, and *P*. *beijingensis* GtfD. The reaction mixtures were incubated for 24 h at 37 °C and pH 5.5 (*L*. *reuteri* NCC 2613 GtfB), pH 5.0 (*L*. *reuteri* 121 GtfB) and pH 7.0 (*P*. *beijingensis* GtfD). (**a**) ^1^H NMR spectrum (D_2_O, 298 K) of the generated products. The anomeric signals indicated as Gα/β and Rα/β correspond to free glucose and reducing -(1 → 4)-D-Glc*p* units, respectively. Chemical shifts are shown in parts per million (ppm) relative to the signal of internal acetone (δ 2.225). (**b**) HPSEC molecular mass distribution of the reaction products formed.

To study the product specificity of the *L*. *reuteri* NCC 2613 GtfB-ΔN in more detail, the products synthesized from amylose V were analysed by one-dimensional ^1^H NMR spectroscopy. As shown in Fig. 5a, ^1^H NMR analysis revealed the presence of two broad anomeric signals indicative of (α1 → 4) linkages (δ ~ 5.40–5.35) and (α1 → 6) linkages (δ ~ 4.97); thus *L*. *reuteri* NCC 2613 GtfB-ΔN also acts as a 4,6-α-GTase. Small signals corresponding to free glucose units (Gα H-1, δ 5.225; Gβ H-1, δ 4.637) and 4-substituted reducing-end glucose residues (Rα H-1, δ 5.225; Rβ H-1, δ 4.652) were detected as well, indicating that trace amounts of glucose, maltose and other small oligosaccharides were also present in this product mixture. This ^1^H NMR spectrum was highly similar to those of the reuteran-like polymers synthesized by the *A*. *chroococcum* GtfD (not shown) and *P*. *beijingensis* GtfD, as indicated by the presence of extra signals strongly overlapping in the (α1 → 4) anomeric region (Fig. 5a). Note that these signals are not present in the NMR spectrum of the IMMP generated by *L*. *reuteri* 121 GtfB (Fig. 5a). The molar ratio of the (α1 → 4)-linked, (α1 → 6)-linked and reducing glucose residues for *L*. *reuteri* NCC 2613 GtfB-ΔN amylose-derived products was 78:22: < 1, also resembling those obtained by incubation of amylose with the *A*. *chroococcum* GtfD (72:26:2) and *P*. *beijingensis* GtfD enzymes (75:25: < 1)^20, 21^. The *L*. *reuteri* 121 GtfB IMMP product showed a significantly larger number of (α1 → 6) linkages (80%).

The amylose-derived products from *L*. *reuteri* NCC 2613 GtfB-ΔN were also analyzed by HPSEC with multidetection. The HPSEC profile of the original amylose V substrate consisted of a single peak eluting at ~21 ml with an average *M*_w_ of 200 × 10^3^ Da. As shown in Fig. 5b, the action of the *L*. *reuteri* NCC 2613 GtfB-ΔN on amylose V resulted in the formation of a peak at a higher elution volume (~29 ml) corresponding to a low molecular mass α-glucan with an average *M*_w_ of 7 × 10^3^ Da, together with a small shoulder peak corresponding to maltose. This HPSEC profile significantly differs from those reported for other 4,6-α-GTases^20, 21^ producing higher molecular mass polymers from amylose V. For example, the *M*_w_ value of the α-glucan generated by *L*. *reuteri* NCC 2613 GtfB-ΔN is only half that of the IMMP products of *L*. *reuteri* 121 GtfB (15 × 10^3^ Da), and it is much smaller than the HMM polysaccharide synthesized by the *A*. *chroococcum* GtfD, with an average *M*_w_ of 13 × 10^6^ Da^20^. The *L*. *reuteri* NCC2613 GtfB-ΔN product profile is also different from that of *P*. *beijingensis* GtfD which showed a bimodal polymer distribution containing both HMM (27 × 10^6^ Da) and LMM (19 × 10^3^ Da) polymers (Fig. 5b **)**. It is also smaller than the reuteran product synthesized by the glucansucrase GtfA from *L*. *reuteri* 121 (45 × 10^3^ Da)^36^.

### Structural characterization of the *L*. *reuteri* NCC 2613 GtfB-ΔN LMM polysaccharide

The amylose-derived reaction mixture was subjected to Bio-Gel P-2 size-exclusion chromatography, and the structural characteristics of the isolated *L*. *reuteri* NCC 2613 GtfB-ΔN LMM polysaccharide were investigated by 1D 2D NMR and methylation analysis (Table 1). As in the case of the reuteran type of polymers^20, 21, 37^, the typical chemical shift values corresponding to consecutive (α1 → 6) linkages were not identified in the 2D NMR spectra of this *L*. *reuteri* NCC 2613 GtfB-ΔN polymer (Fig. S2). Taken together, these analyses confirmed that similar to GtfD type of enzymes, the *L*. *reuteri* NCC 2613 GtfB-ΔN synthesizes a reuteran-like α-glucan, providing the first evidence of this product specificity within the GtfB-like GH70 subfamily. The structural characteristics of the different amylose-derived reuteran type of polymers are summarized in Table 1. Regarding its size and (α1 → 4):(α1 → 6) linkage ratio, the α-glucan synthesized by the *L*. *reuteri* NCC 2613 GtfB-ΔN resembles mostly the LMM *P*. *beijingesis* GtfD polymer, however, it contains higher amounts of alternating (α1 → 4)/(α1 → 6) glycosidic linkages as indicated by the increased amount of 6-substituted glucopyranosyl units (i.e. 10% rather than 5%).

**Table 1:** Structural characterization of the polysaccharide formed upon incubation of amylose V with the *L*. *reuteri* NCC 2613 GtfB-ΔN enzyme.

Parameter	Type of glucosyl units	*A*. *chroococcum* GtfD polymer^**c**^	*P*. *beijingensis* GtfD HMM polymer^**d**^	*P*. *beijingensis* GtfD LMM polymer^**d**^	*L*. *reuteri* NCC 2613 GtfB-ΔN polymer
Methylation analysis (%)	Glc*p*(1 →	19	17	15	15
	→ 4)-Glc*p*-(1 →	45	54	62	59
	→ 6)-Glc*p*-(1 →	18	11	5	10
	→ 4,6)-Glc*p*-(1 →	18	18	18	16
NMR chemical shift (%)^**a**^	(α1 → 4)	68	71	77	75
	(α1 → 6)	32	29	23	25
Molecular mass (10^3^ Da)^**b**^		13 10^3^	27 10^3^	19	7

For comparison the characteristics of the polymers produced by the *A*. *chroococcum* and *P*. *beijingensis* GtfD 4,6-α-GTases are included as well.

^a^The data represents the ratios of integration of the surface areas of the (α1 → 6) linkage signal at 4.97 ppm and the (α1 → 4) linkage signal at 5.36 ppm in the ^1^H NMR spectra of the polysaccharides (see Fig. S2). ^b^The average molecular mass of polysaccharide was determined in duplicate. ^c^Taken from Gangoiti *et al*.^20^. ^d^Taken from Gangoiti *et al*. ^21^.

### Oligosaccharides formed from maltoheptaose in time by the *L*. *reuteri* NCC 2613 GtfB-ΔN

**Figure 6:**
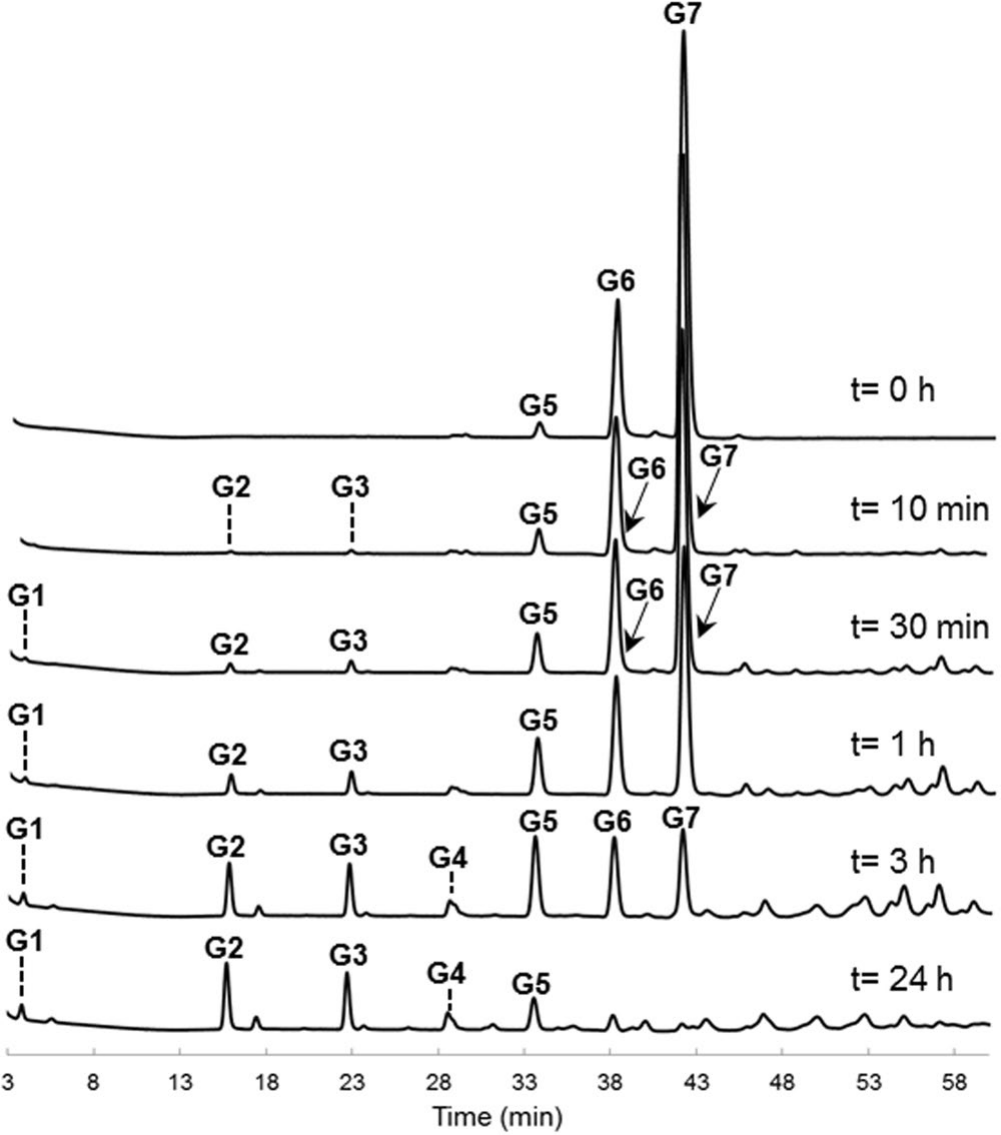
HPAEC-PAD profile of the oligosaccharide mixture formed upon the incubation of *L*. *reuteri* NCC 2613 GtfB-ΔN (20 μg ml^−1^) with maltoheptaose for t = 1 h, 3 h and 24 h (pH 5.5, 37 °C). The identity of peaks was assigned using commercial oligosaccharide standards. G1, glucose; G2–G7, maltose to maltoheptaose; iso-G2, isomaltose; and iso-G3, isomaltotriose.

To gain a better understanding of the *L*. *reuteri* NCC 2623 GtfB-ΔN reuteran-like product formation, the oligosaccharides formed from maltoheptaose in time were analyzed by HPAEC (Fig. 6). From maltoheptaose (slightly contaminated with maltohexaose and maltopentaose), the *L*. *reuteri* NCC 2613 GtfB-ΔN released maltose, maltotriose and maltopentaose as the main hydrolysis products, at the early stage of the reaction. Together with these first clear hydrolysis products, a significant number of peaks eluted at higher elution times than the maltooctaose standard (elution time = 45.7 min) with products resulting from its transglycosylating activity. After 24 h of reaction, the maltoheptaose substrate was completely depleted, whereas some MOS of low DP and oligosaccharides of unknown structure remained in the reaction mixture. Notably, only trace amounts of glucose were detected during the 24 h of reaction. The formation of maltose and maltotriose as main hydrolysis products, combined with the appearance of peaks corresponding to oligosaccharides with DP higher than 8, suggests that the *L*. *reuteri* NCC 2613 GtfB enzyme preferentially transfers MOS of different DP (instead of glucose) to another glucan chain to form a reuteran-like polymer. This mechanism of polymerization differs from the one observed for GSs, which only transfer a single glucosyl unit per reaction cycle^10, 11, 26^. Instead, the mode of action of the *L*. *reuteri* NCC 2613 GtfB-ΔN resembles that of GtfD 4,6-α-GTase which also produces reuteran-type polymers^20, 21^. Similarly, the *L*. *fermentum* GtfB 4,3-α-GTase converts amylose into a polymer containing alternating (α1 → 3)/(α1 → 4) linkages and (α1 → 3,4) branching points by transferring MOS of different DPs^18^. Whereas in GSs the active site is blocked beyond subsite -1, the GH70 starch-active enzymes appear to have more than one donor substrate binding subsite, allowing the elongation process to occur by successive transfer of MOS units derived from starch. Indeed, the recent elucidation of the *L*. *reuteri* 121 GtfB-ΔNΔV 4,6-α-GTase structure revealed that its active site presents multiple donor binding subsites similar to the evolutionary related GH13 family proteins^17^.

### *L*. *reuteri* NCC 2613 GtfB-ΔN acceptor substrate reaction studies

**Figure 7:**
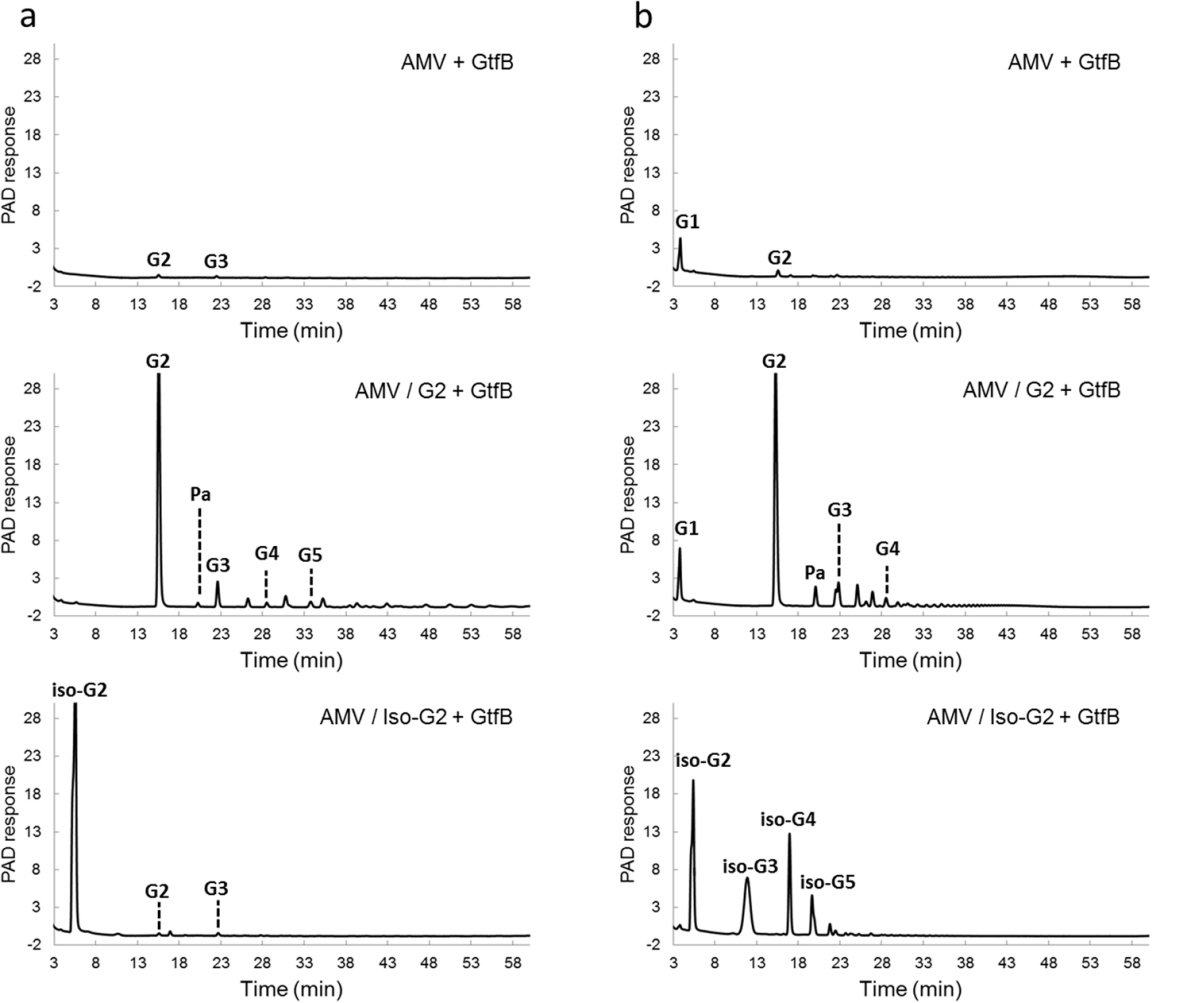
HPAEC-PAD profiles of the oligosaccharide mixtures generated by incubating the *L*. *reuteri* NCC 2613 GtfB-ΔN enzyme (40 μg ml^−1^) (**a**) and *L*. *reuteri* 121 GtfB enzyme (40 μg ml^−1^) (**b**) with 0.35% amylose V (AMV) (donor substrate) or amylose V with 25 mM maltose or 25 mM isomaltose (acceptor substrates) for 24 h at 37 °C. The identity of peaks was assigned using commercial oligosaccharide standards. G1, glucose; G2–G4, maltose to maltotetraose; iso-G2-iso-G5, isomaltose to isomaltopentaose; Pa, panose.

The *L*. *reuteri* NCC 2613 GtfB-ΔN and *L*. *reuteri* 121 GtfB enzymes were incubated with amylose V as donor substrate in the presence or absence of maltose and isomaltose as acceptor substrates for 24 h. As depicted in Fig. 7a, maltose served as acceptor substrate for *L*. *reuteri* NCC 2613 GtfB-ΔN, resulting in formation of panose, maltotriose, maltotetraose and maltopentaose, and significant amounts of other unidentified oligosaccharides. The *L*. *reuteri* 121 GtfB displayed a different product distribution, but this enzyme was also able to use maltose as an acceptor substrate, yielding panose, maltotriose and maltotetraose, together with a series of elongated products with successive (α1 → 6) linkages and increasing degrees of polymerization (Fig. 7b). These results show that both GtfB enzymes elongate maltose forming either a new (α1 → 4) or (α1 → 6) linkage. Acceptor reactions with isomaltose more clearly reflected the different modes of action of these GtfB enzymes. The *L*. *reuteri* 121 GtfB enzyme clearly preferred isomaltose as acceptor substrate over maltose, as indicated by the detection of significant amounts of isomaltotriose, isomaltotetraose and isomaltopentaose (resulting from the elongation of the isomaltose by successive (α1 → 6) linkages). In contrast, no significant change in oligosaccharide formation was observed when amylose V was incubated with *L*. *reuteri* NCC 2613 GtfB-ΔN in the presence or absence of isomaltose. Thus, whereas the *L*. *reuteri* 121 GtfB preferentially elongates oligosaccharides with α1 → 6 linked non-reducing ends, the *L*. *reuteri* NCC 2613 GtfB-ΔN is unable to recognize isomaltose as an acceptor substrate, similar to *A*. *chroococcum* GtfD^20^. In agreement with these observations, the *L*. *reuteri* NCC 2613 GtfB-ΔN and *L*. *reuteri* 121 GtfB products differ by the absence or presence of consecutive (α1 → 6) linkages in their structures, respectively.

### Enzymatic hydrolysis of the *L*. *reuteri* NCC 2613 GtfB-ΔN reuteran-like polysaccharide

**Figure 8:**
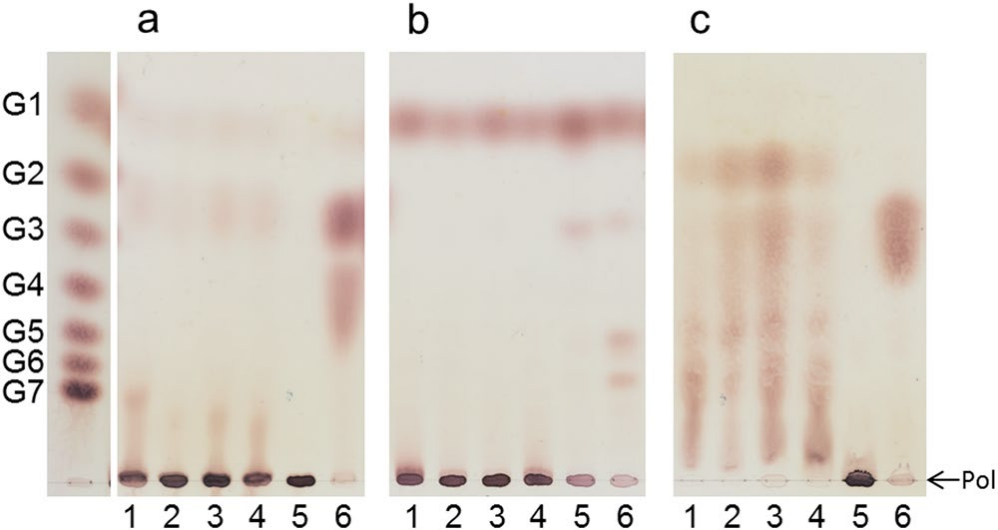
TLC analysis of the *L*. *reuteri* NCC 2613 GtfB-ΔN polysaccharide (5 mg ml^−1^) after digestion with excess amounts of (**a**) *Aspergillus oryzae* α-amylase, (**b**) *Chaetomium erraticum* dextranase and (**c**) *Klebsiella planticola* pullulanase M1 for 48 h at 37 °C. For comparison, the reuteran-like polymers produced by *A*. *chroococcum* NCIMB 8003 GtfD and *P*. *beijngensis* GtfD, and the IMMP product (~95% (α1 → 6) linkages) synthesized by *L*. *reuteri* 121 GtfB were subjected to the same enzymatic treatments. Lanes 1–5: reaction products generated by the enzymatic treatment of the *L*. *reuteri* NCC 2613 GtfB-ΔN polymer, *A*. *chroococcum* GtfD polymer, *P*. *beijingensis* GtfD HMM polymer, *P*. *beijingensis* GtfD LMM polymer, and *L*. *reuteri* 121 GtfB polymer, respectively. Lane 6, positive controls for the α-amylase, dextranase and pullulanase digestions: amylose (**a**), dextran (**b**) and pullulan (**c**). Lane S, standard: glucose (G1) to maltoheptaose (G7); Pol, polymer.

**Figure 9:**
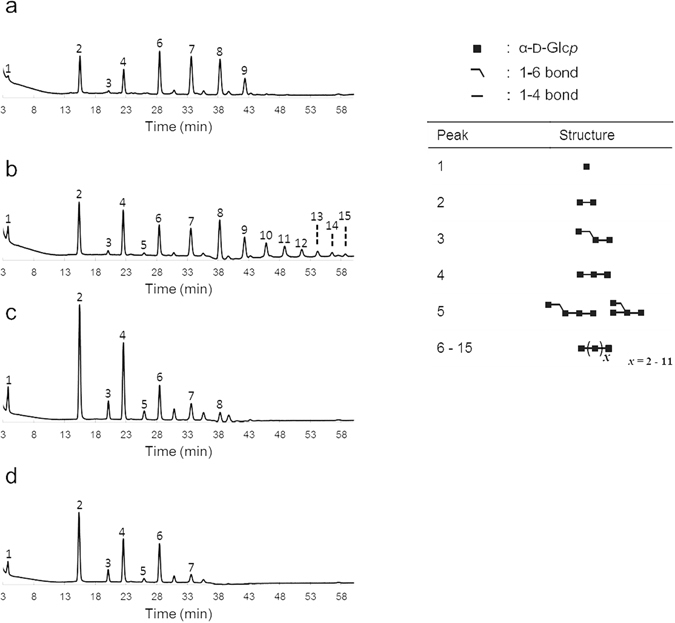
HPAEC-PAD profile of the oligosaccharides formed by digesting the *L*. *reuteri* NCC 2613 GtfB polymer (**a**), *P*. *beijingensis* GtfD LMM polymer (**b**), *P*. *beijingensis* GtfD HMM polymer (**c**), and *A*. *chroococcum* GtfD polymer (D) using pullulanase M1. Established oligosaccharide structures are included. The identity of peaks 1–15 was assigned using commercial oligosaccharide standards and by comparison with the profile of the pullulanase hydrolysate of reuteran^37^.

The reuteran-like structure of the α-glucan produced by *L*. *reuteri* NCC 2613 GtfB-ΔN was further confirmed by treating this α-glucan with excess amounts of different hydrolytic enzymes: α-amylase, dextranase and pullulanase. For comparison, the IMMP synthesized by the *L*. *reuteri* 121 GtfB 4,6-α-GTase and the reuteran-like polymers produced by the *A*. *chroococcum* and *P*. *beijingensis* GtfD 4,6-α-GTases were subjected in parallel to the same enzymatic treatments. As shown in Fig. 8, the *L*. *reuteri* NCC 2613 GtfB-ΔN polymer was quite resistant to the endo-(1 → 4) hydrolase activity of the α-amylase. Compared to the amylose control that was completely degraded, only trace amounts of HMM oligosaccharides and maltose were formed when this polymer was incubated with the α-amylase. Similar hydrolytic patterns were obtained for the reuteran-like polymers synthesized by the *A*. *chroococcum* and *P*. *beijingensis* GtfD 4,6-α-GTases, whereas these small amounts of maltose or other oligosaccharides were not detected in case of IMMP incubation with α-amylase. The *L*. *reuteri* NCC 2613 GtfB-ΔN polymer was also subjected to dextranase and pullulanase M1 enzymatic hydrolysis, which catalyzes the hydrolysis of (α1 → 6) glycosidic linkages. Whereas dextranase specifically attacks linear sequences of (α1 → 6)-linked D-glucopyranosyl repeating units, pullulanase is specific for α1 → 6 linkages in the backbone chains of pullulan and at branching points of starch molecules. For the dextranase and pullulanase enzymatic treatments, dextran and pullulan were used as positive controls, respectively. As expected, the *L*. *reuteri* NCC 2613 GtfB-ΔN polymer and the *A*. *chroococcum* and *P*. *beijingensis* GtfD polymers were not degraded by the action of dextranase, instead these polymers were hydrolyzed by pullulanase. Whereas pullulan digestion by pullulanase results in the production of maltotriose, multiple low molecular mass products are obtained in the case of these reuteran-like polymers, reflecting the presence of linear (α1 → 4) sequences of different DP in their structure (See Fig. 9 for a more detailed analysis). In contrast, the IMMP product was resistant to the pullulanase treatment, but it was digested by the endo-(α1 → 6)-hydrolase activity of dextranase. These results are in agreement with the presence of only successive (α1 → 6) linkages in the *L*. *reuteri* 121 GtfB polymer and their absence in the *L*. *reuteri* NCC 2613 GtfB-ΔN polymer. Similar to the reuteran type of polymers synthesized by GtfA GS and GtfD 4,6-α-GTases, and differing from IMMP, this *L*. *reuteri* NCC 2613 GtfB-ΔN polymer appears to contain alternating (α1 → 6)/(α1 → 4) linkages and (α1 → 4,6) branching points.

**Figure 10:**
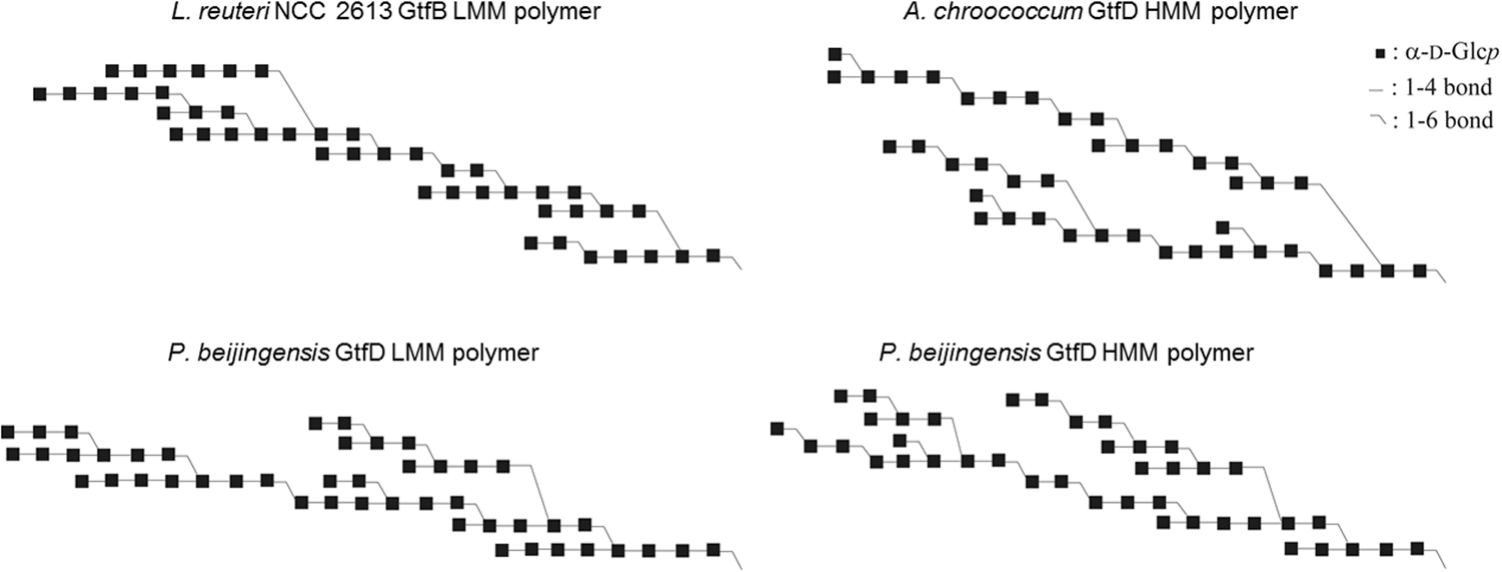
Visual representation of composite structures for the *L*. *reuteri* NCC 2613 GtfB-ΔN LMM polymer, the *A*. *chroococcum* NCIMB 8003 GtfD HMM polymer^20^, and the HMM and LMM *P*. *beijingensis* GtfD polymers^21^ formed from amylose V. The composite structures contain all structural features established for the respective products. Quantities of each structural element fit with the combined data of 1D ^1^H NMR integration and methylation analysis, as well as enzymatic degradation studies with pullulanase.

Further information about the structure of the *L*. *reuteri* NCC 2613 GtfB-ΔN polymer was obtained by the identification of the reaction products that resulted from the pullulanase treatment by HPAEC. As shown in Fig. 9a, the pullulanase digest of the *L*. *reuteri* NCC 2613 GtfB-ΔN polymer, yielded mainly glucose, and a mixture of MOS from DP2 to 7. This finding indicates that this polymer is formed by maltose, maltotriose, maltotetraose, maltopentaose, maltohexaose and maltoheptaose elements linked by single (α1 → 6) linkages. These structural elements are also present in the LMM *P*. *beijingensis* GtfD polymer, however with small amounts of longer linear (α1 → 4) sequences (from DP8 to DP13) also being detected (Fig. 9b). As reported before, pullulanase degraded the HMM reuteran polymers synthesized by *P*. *beijingensis* GtfD and *A*. *chroococcum* GtfD enzymes into MOS units up to DP6 and DP5, respectively (Fig. 9c and d)^20^. Overall, these HPAEC profiles suggest that the 4,6-α-GTases characterized so far have a preference for transferring different lengths of (α1 → 4) glucan chains, yielding as a result, reuteran polymers with unique structures. Figure 10 shows composite models for the reuteran-like polymers produced by the *L*. *reuteri* NCC2613 GtfB-ΔN and the previously characterized GtfD type of enzymes. The *L*. *reuteri* NCC2613 GtfB-ΔN enlarges the variety of reuteran-like α-glucans that can be easily synthesized using GH70 enzymes from amylose.

## Conclusions

In this work a novel GH70 4,6-α-GTase enzyme catalyzing the formation of a reuteran-like polymer from amylose was identified in the NCC genome database containing the sequences of more than 2700 isolates, composed mainly of lactic acid bacteria. This enzyme is encoded by *L*. *reuteri* NCC 2613 and clearly belongs to the GtfB-like GH70 subfamily, but it displays a reaction and product specificity resembling that described for the recently characterized GtfD type of enzymes also producing reuteran-like polymers from maltodextrin/starch substrates. The more open architecture of the *L*. *reuteri* NCC 2613 GtfB-ΔN active site may explain its ability to synthesize branched products, whereas the *L*. *reuteri* 121 GtfB 4,6-α-GTase, due to the presence of a tunnel, only forms linear products. Based on *in vitro* digestibility studies, it appears likely that these reuteran type of polymers are not- or only slowly digested by human gastrointestinal tract enzymes, opening new perspectives for the application of these enzymes for the reduction of calories and glycemic index of starchy products^21^. In contrast to GtfD enzymes producing bacteria, *L*. *reuteri* NCC 2613 possesses the Generally Recognised As Safe (GRAS) status, providing an advantage for its use by the food industry. Overall, this study represents a good example of how to exploit large-scale sequence data to identify new biotechnologically relevant enzymes. Clearly, the *L*. *reuteri* NCC 2613 GtfB, and its homologs encoded by *L*. *reuteri* strains NCC 2592, NCC 2603, and NCC 3072 represent new evolutionary intermediates between GH13 and GH70 families. Besides, the *L*. *reuteri* NCC 2613 GtfB enzyme is a valuable biocatalyst for the conversion of starch present in food into soluble non-digestible fibers that afford a reduced release of glucose in the human digestive tract. The characterization of this enzyme, showing unique variations in some of the residues forming the substrate binding residues in the active site of GS enzymes, has provided new insights into the understanding of the structural features determining product specificity in the GtfB GH70 subfamily.

## Electronic supplementary material

Supplementary Information
