# Supplementary material for: Mining novel starch-converting Glycoside Hydrolase 70 enzymes from the Nestlé Culture Collection genome database: The Lactobacillus reuteri NCC 2613 GtfB
Source: Sci Rep. 2017 Aug 30;7:9947. doi: 10.1038/s41598-017-07190-z (PMC5577214; doi:10.1038/s41598-017-07190-z)

Supplementary Information

Mining novel starch-converting Glycoside Hydrolase 70 enzymes from the Nestlé Culture Collection genome database: The *Lactobacillus reuteri* NCC 2613 GtfB

Joana Gangoiti [a, d], Sander S. van Leeuwen [a], Xiangfeng Meng [a], Stéphane Duboux [b], Christina Vafiadi [b], Tjaard Pijning [c], Lubbert Dijkhuizen [a *]

[a] Microbial Physiology, Groningen Biomolecular Sciences and Biotechnology Institute (GBB), University of Groningen, Nijenborgh 7, 9747 AG Groningen, The Netherlands

[b] Nestlé Research Center, Vers-Chez-Les-Blanc, Lausanne, Switzerland

[c] Biophysical Chemistry, Groningen Biomolecular Sciences and Biotechnology Institute (GBB), University of Groningen, Nijenborgh 7, 9747 AG Groningen, The Netherlands

[d] Current address: J. Gangoiti, CarbExplore Research BV, Zernikepark 12, 9747 AN Groningen, The Netherlands

[*] Corresponding author

E-mail address: L.Dijkhuizen@rug.nl (L. Dijkhuizen)

Key words: 4,6-α-glucanotransferase, *Lactobacillus reuteri*, Glucansucrase, Family GH70, reuteran, α-Glucan

SUPPLEMENTARY TABLE

SUPPLEMENTARY FIGURES

Table S1. GenBank accession numbers of the family GH70 protein sequences used in the phylogenetic tree of Figure 1.

Accession number	Bacterium strain
AAC43483.1	*Streptococcus gordonii* str. Challis substr. CH1
BAA26114.1	*Streptococcus mutans*
BAC07265.1	*Streptococcus sobrinus* B13N
KY595679	*Lactobacillus delbrueckii* NCC 119
KY595675	*Lactobacillus delbrueckii* NCC 147
KY595681	*Lactobacillus delbrueckii* NCC 1579
KY595680	*Lactobacillus delbrueckii* NCC 164
KY595682	*Lactobacillus delbrueckii* NCC 167
KY595683	*Lactobacillus delbrueckii* NCC 169
KY595684	*Lactobacillus delbrueckii* NCC 188
KY595685	*Lactobacillus delbrueckii* NCC 2506
KY595688	*Lactobacillus delbrueckii* NCC 2510
KY595686	*Lactobacillus delbrueckii* NCC 2574
KY595687	*Lactobacillus delbrueckii* NCC 2595
KY595689	*Lactobacillus delbrueckii* NCC 2727
KY595690	*Lactobacillus delbrueckii* NCC 2812
KY595691	*Lactobacillus delbrueckii* NCC 3035
KY595692	*Lactobacillus delbrueckii* NCC 3077
KY595678	*Lactobacillus delbrueckii* NCC 32
KY595693	*Lactobacillus delbrueckii* NCC 39
KY595699	*Lactobacillus delbrueckii* NCC 56
KY595694	*Lactobacillus delbrueckii* NCC 590
KY595695	*Lactobacillus delbrueckii* NCC 601
KY595698	*Lactobacillus delbrueckii* NCC 603
KY595696	*Lactobacillus delbrueckii* NCC 610
KY595697	*Lactobacillus delbrueckii* NCC 612
KY595700	*Lactobacillus delbrueckii* NCC 618
KY595704	*Lactobacillus delbrueckii* NCC 621
KY595702	*Lactobacillus delbrueckii* NCC 627
KY595701	*Lactobacillus delbrueckii* NCC 636
KY595703	*Lactobacillus delbrueckii* NCC 637
KY595705	*Lactobacillus delbrueckii* NCC 648
KY595706	*Lactobacillus delbrueckii* NCC 653
KY595707	*Lactobacillus delbrueckii* NCC 657
KY595708	*Lactobacillus delbrueckii* NCC 667
KY595709	*Lactobacillus delbrueckii* NCC 675
KY595710	*Lactobacillus delbrueckii* NCC 687
KY595711	*Lactobacillus delbrueckii* NCC 696
KY595712	*Lactobacillus delbrueckii* NCC 706
KY595714	*Lactobacillus delbrueckii* NCC 707
KY595713	*Lactobacillus delbrueckii* NCC 710
KY595715	*Lactobacillus delbrueckii* NCC 716
KY595677	*Lactobacillus delbrueckii* NCC 71
KY595716	*Lactobacillus delbrueckii* NCC 723
KY595717	*Lactobacillus delbrueckii* NCC 731
KY595674	*Lactobacillus delbrueckii* NCC 749
KY595718	*Lactobacillus delbrueckii* NCC 800
KY595719	*Lactobacillus delbrueckii* NCC 801
KY595720	*Lactobacillus delbrueckii* NCC 828
KY595721	*Lactobacillus delbrueckii* NCC 829
KY595676	*Lactobacillus delbrueckii* NCC 82
KY595722	*Lactobacillus delbrueckii* NCC 853
KY595723	*Lactobacillus delbrueckii* NCC 861
KY595724	*Lactobacillus delbrueckii* NCC 865
KY595725	*Lactobacillus delbrueckii* NCC 875
KY595726	*Lactobacillus delbrueckii* NCC 895

KY595727	*Lactobacillus delbrueckii* NCC 902
KY595728	*Lactobacillus delbrueckii* NCC 924
KY595731	*Lactobacillus delbrueckii* NCC 946
KY595729	*Lactobacillus delbrueckii* NCC 960
KY595730	*Lactobacillus delbrueckii* NCC 968
AOR73699	*Lactobacillus fermentum* NCC 2970
KY595732	*Lactobacillus fermentum* NCC 3053
KY595733	*Lactobacillus fermentum* NCC 3057
KY595734	*Lactobacillus fermentum* NCC 3064
KY595737	*Lactobacillus fermentum* NCC 3065
KY595735	*Lactobacillus fermentum* NCC 515
KY595736	*Lactobacillus fermentum* NCC 528
KY595738	*Lactobacillus fermentum* NCC 729
KY595739	*Lactobacillus fermentum* NCC 743
KY595741	*Lactobacillus plantarum* NCC 1287
KY595740	*Lactobacillus plantarum* NCC 1323
KY595742	*Lactobacillus plantarum* NCC 1517
KY595743	*Lactobacillus plantarum* NCC 1690
KY595744	*Lactobacillus plantarum* NCC 1736
KY595745	*Lactobacillus plantarum* NCC 2502
KY595748	*Lactobacillus plantarum* NCC 79
KY595673	*Lactobacillus plantarum* NCC 81
KY595749	*Lactobacillus plantarum* NCC 84
KY595746	*Lactobacillus reuteri* NCC 1945
KY595747	*Lactobacillus reuteri* NCC 2592
KY595750	*Lactobacillus reuteri* NCC 2603
KY595751	*Lactobacillus reuteri* NCC 2613
KY595752	*Lactobacillus reuteri* NCC 2884
KY595753	*Lactobacillus reuteri* NCC 2885
KY595754	*Lactobacillus reuteri* NCC 3062
KY595756	*Lactobacillus reuteri* NCC 3072
KY595755	*Lactobacillus sanfranciscensis* NCC 2505
KY595757	*Lactobacillus sanfranciscensis* NCC 2512
KY595758	*Lactobacillus sanfranciscensis* NCC 2532
KY595759	*Lactobacillus sanfranciscensis* NCC 2550
KY595760	*Lactobacillus sanfranciscensis* NCC 2558
KY595761	*Lactobacillus sanfranciscensis* NCC 2572
KY595762	*Lactobacillus sanfranciscensis* NCC 2580
KY595763	*Lactobacillus sanfranciscensis* NCC 2602
KY595764	*Lactobacillus sanfranciscensis* NCC 2607
KY595765	*Lactobacillus sanfranciscensis* NCC 2615
KY595766	*Lactobacillus sanfranciscensis* NCC 2629
KY595767	*Lactobacillus sanfranciscensis* NCC 2673
KY595768	*Lactobacillus sanfranciscensis* NCC 2678
KY595769	*Lactobacillus sanfranciscensis* NCC 2681
KY595770	*Lactobacillus sanfranciscensis* NCC 2694
KY595771	*Lactobacillus sanfranciscensis* NCC 2731
KY595772	*Lactobacillus sanfranciscensis* NCC 2764
KY595773	*Lactobacillus sanfranciscensis* NCC 2769
KY595774	*Lactobacillus sanfranciscensis* NCC 463
KY595776	*Leuconostoc citreum* NCC 2514
KY595775	*Streptococcus thermophilus* NCC 2408
KY595777	*Streptococcus thermophilus* NCC 903
AAU08014.2	*Lactobacillus reuteri* 121
ACB62096.1	*Exiguobacterium sibiricum* (strain DSM 17290 / JCM 13490 / 255-15)
AJE22990.1	*Azotobacter chroococcum* NCIMB 8003
WP_052702730.1	*Paenibacillus beijingensis*
CCK33644.1	*Lactobacillus animalis*
CCK33643.1	*Lactobacillus curvatus*
AAU08008.1	*Lactobacillus fermentum*
AAU08006.1	*Lactobacillus parabuchneri*

AAU08015.1	*Lactobacillus reuteri*
AAU08001.1	*Lactobacillus reuteri*
AAY86923.1	*Lactobacillus reuteri*
AAU08004.1	*Lactobacillus reuteri*
ABP88726.1	*Lactobacillus reuteri*
AAU08011.1	*Lactobacillus sakei*
AIM52834.1	*Leuconostoc citreum*
ACY92456.2	*Leuconostoc citreum*
BAF96719.1	*Leuconostoc citreum*
ACA83218.1	*Leuconostoc citreum (strain KM20)*
AAB95453.1	*Leuconostoc mesenteroides*
ACT20911.1	*Leuconostoc lactis*
ABC75033.1	*Leuconostoc mesenteroides*
ABF85832.1	*Leuconostoc mesenteroides*
CAB76565.1	*Leuconostoc mesenteroides*
AAG38021.1	*Leuconostoc mesenteroides*
AAS79426.1	*Leuconostoc mesenteroides*
AFP53921.1	*Leuconostoc mesenteroides*
AAQ98615.2	*Leuconostoc mesenteroides*
AAG61158.1	*Leuconostoc mesenteroides*
CAB65910.2	*Leuconostoc mesenteroides*
AAN38835.1	*Leuconostoc mesenteroides*
AAD10952.1	*Leuconostoc mesenteroides*
BAA90527.1	*Leuconostoc mesenteroides*
BAF62338.1	*Streptococcus criceti*
AAA26898.1	*Streptococcus downei (Streptococcus sobrinus)*
AAC63063.1	*Streptococcus downei (Streptococcus sobrinus)*
AAN58619.1	*Streptococcus mutans* serotype c (strain ATCC 700610 / UA159)
AAN58705.1	*Streptococcus mutans* serotype c (strain ATCC 700610 / UA159)
AAN58706.1	*Streptococcus mutans* serotype c (strain ATCC 700610 / UA159)
BAA95201.1	*Streptococcus oralis*
BAF62337.1	*Streptococcus orisuis*
AAA26896.1	*Streptococcus salivarius*
CAA77898.1	*Streptococcus salivarius*
AAC41412.1	*Streptococcus salivarius*
AAC41413.1	*Streptococcus salivarius*
BAF43788.1	*Streptococcus sanguinis*
BAA14241.1	*Streptococcus downei* (*Streptococcus sobrinus*)
BAA02976.1	*Streptococcus sobrinus*
AAX76986.1	*Streptococcus sobrinus*
ACK38203.1	*Weissella cibaria*
ADB43097.3	*Weissella cibaria*
AKE50934.1	*Weissella confusa* (*Lactobacillus confusus*)
CCF30682.1	*Weissella confusa* LBAE C39-2
AHU88292.1	*Weissella confusa* (*Lactobacillus confusus*)
CDX66641.1	*Leuconostoc citreum* NRRL B-1299
CDX66896.1	*Leuconostoc citreum* NRRL B-1299
CDX66895.1	*Leuconostoc citreum* NRRL B-1299
CDX66820.1	*Leuconostoc citreum* NRRL B-1299
CDX67012.1	*Leuconostoc citreum* NRRL B-1299

FIG. S1. SDS-PAGE analysis of the recombinant *L. reuteri* NCC 2613GtfB protein purified from *E. coli* BL21 Star (DE3). Lane M, molecular mass standards; lanes 1 and 2, samples of cell free extract; lane 3, sample of the insoluble fraction after centrifugation of lysed cells; lane 4, pooled fractions after Ni-NTA agarose column chromatography. Bands corresponding to the GtfB protein are marked with an arrow.

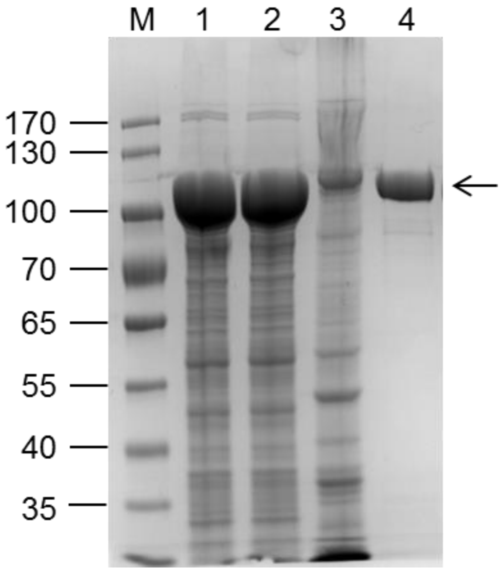

FIG. S2. 500-MHz 1D ^{1}H NMR spectrum, 2D ^{1}H-^{1}H TOCSY spectra (mixing time 150 ms), and 2D ^{13}C-^{1}H HSQC spectrum (D_2O, 298K) of the α-glucan generated by the *L. reuteri* NCC 2613 GtfB-ΔN enzyme, isolated by size-exclusion chromatography on a Biogel P2 column. The reaction products were obtained from 0.6% (w v^{-1}) amylose V, incubated with 40 µg ml^{-1} of the *L. reuteri* NCC 2613 GtfB-ΔN enzyme for 24 h at 37°C and pH 5.5. Peaks for (α1→4) and (α1→6) anomeric signals have been indicated. Structural reporter peaks a: H-4 for 6-substituted Glc*p*, b: H-4 for terminal Glc*p*, c: for H-4 for 4-substituted Glc*p*, d: H-6a for 6-substituted Glc*p* and e: H-6b for 6-substituted Glc*p*.

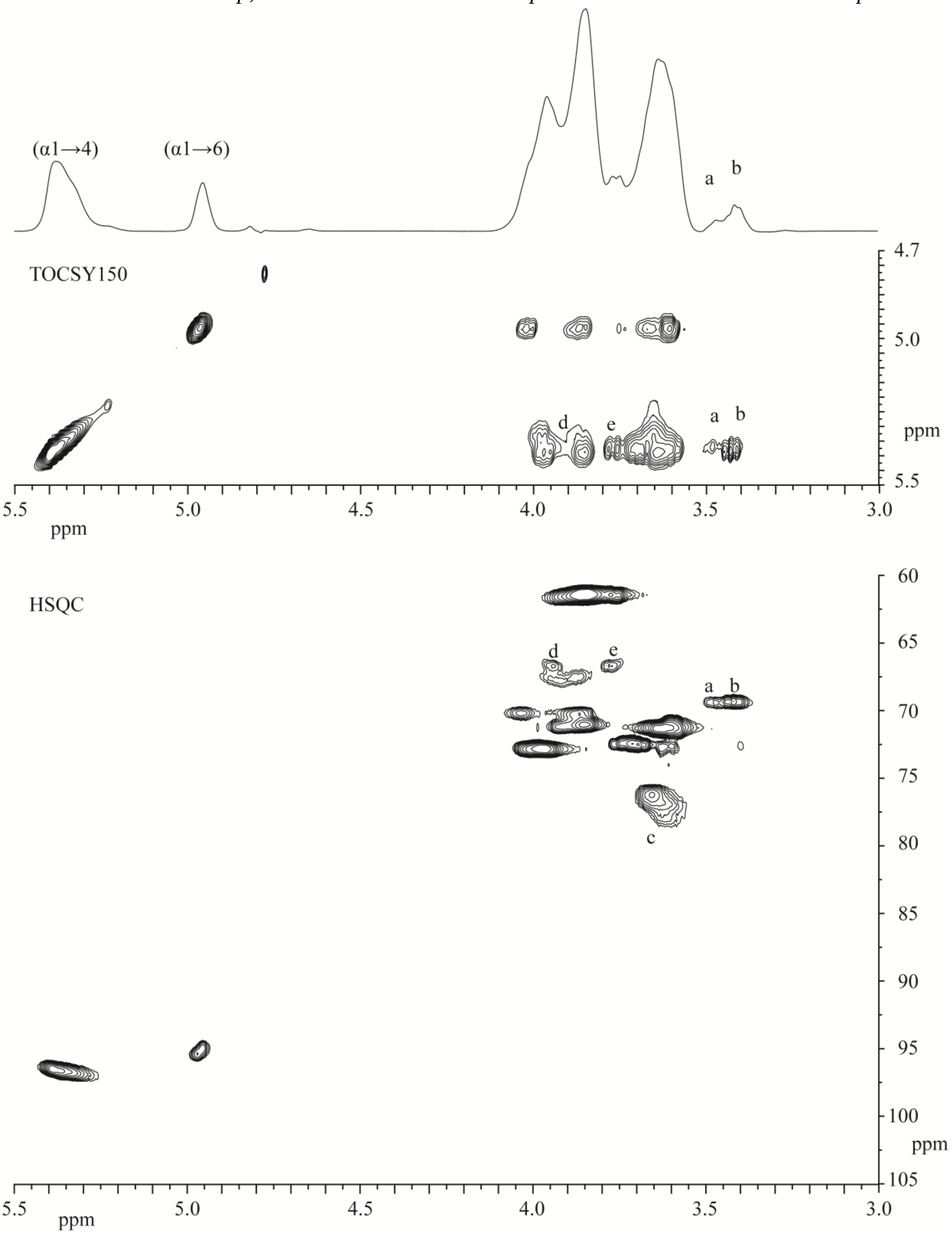

Supplement: Supplementary file 1 — Supplementary Information [file 41598_2017_7190_MOESM1_ESM.pdf]
